# Removal of a Membrane Anchor Reveals the Opposing Regulatory Functions of Vibrio cholerae Glucose-Specific Enzyme IIA in Biofilms and the Mammalian Intestine

**DOI:** 10.1128/mBio.00858-18

**Published:** 2018-09-04

**Authors:** Vidhya Vijayakumar, Audrey S. Vanhove, Bradley S. Pickering, Julie Liao, Braden T. Tierney, John M. Asara, Roderick Bronson, Paula I. Watnick

**Affiliations:** a Division of Infectious Diseases, Boston Children’s Hospital, Harvard Medical School, Boston, Massachusetts, USA; b Department of Biomedical Informatics, Harvard Medical School, Boston, Massachusetts, USA; c Department of Microbiology and Immunobiology, Harvard Medical School, Boston, Massachusetts, USA; d Division of Signal Transduction, Beth Israel Deaconess Medical Center, Boston, Massachusetts, USA; e Department of Medicine, Harvard Medical School, Boston, Massachusetts, USA; University of Washington

**Keywords:** Vibrio cholerae, amphipathic helix, biofilms, phosphoenolpyruvate phosphotransferase system, sugar transport

## Abstract

The Vibrio cholerae phosphoenolpyruvate phosphotransferase system (PTS) is a well-conserved, multicomponent phosphotransfer cascade that coordinates the bacterial response to carbohydrate availability through direct interactions of its components with protein targets. One such component, glucose-specific enzyme IIA (EIIA^Glc^), is a master regulator that coordinates bacterial metabolism, nutrient uptake, and behavior by direct interactions with cytoplasmic and membrane-associated protein partners. Here, we show that an amphipathic helix (AH) at the N terminus of V. cholerae EIIA^Glc^ serves as a membrane association domain that is dispensable for interactions with cytoplasmic partners but essential for regulation of integral membrane protein partners. By deleting this AH, we reveal previously unappreciated opposing regulatory functions for EIIA^Glc^ at the membrane and in the cytoplasm and show that these opposing functions are active in the laboratory biofilm and the mammalian intestine. Phosphotransfer through the PTS proceeds in the absence of the EIIA^Glc^ AH, while PTS-dependent sugar transport is blocked. This demonstrates that the AH couples phosphotransfer to sugar transport and refutes the paradigm of EIIA^Glc^ as a simple phosphotransfer component in PTS-dependent transport. Our findings show that Vibrio cholerae EIIA^Glc^, a central regulator of pathogen metabolism, contributes to optimization of bacterial physiology by integrating metabolic cues arising from the cytoplasm with nutritional cues arising from the environment. Because pathogen carbon metabolism alters the intestinal environment, we propose that it may be manipulated to minimize the metabolic cost of intestinal infection.

## Abstract (precis)

The V. cholerae phosphoenolpyruvate phosphotransferase system (PTS) is a well-conserved, multicomponent phosphotransfer cascade that regulates cellular physiology and virulence in response to nutritional signals. Glucose-specific enzyme IIA (EIIA^Glc^), a component of the PTS, is a master regulator that coordinates bacterial metabolism, nutrient uptake, and behavior by direct interactions with protein partners. We show that an amphipathic helix (AH) at the N terminus of V. cholerae EIIA^Glc^ serves as a membrane association domain that is dispensable for interactions with cytoplasmic partners but essential for regulation of integral membrane protein partners. By removing this amphipathic helix, hidden, opposing roles for cytoplasmic partners of EIIA^Glc^ in both biofilm formation and metabolism within the mammalian intestine are revealed. This study defines a novel paradigm for AH function in integrating opposing regulatory functions in the cytoplasm and at the bacterial cell membrane and highlights the PTS as a target for metabolic modulation of the intestinal environment.

## INTRODUCTION

The phosphoenolpyruvate phosphotransferase system (PTS) is a highly conserved phosphotransfer cascade consisting of the phosphodonor phosphoenolpyruvate, which is a key high-energy intermediate in glycolysis and gluconeogenesis, and four phosphotransfer proteins located in the cytoplasm that are termed enzyme I (EI), histidine protein (HPr), enzyme IIA (EIIA), and enzyme IIB (EIIB) (1). These intermediates regulate distinct aspects of cellular physiology, often as a function of their phosphorylation state, through direct interactions with protein partners. A paralogous family of PTS-associated carbohydrate-specific transporters, termed enzymes IIC (EIICs), do not participate directly in the PTS phosphotransfer cascade. Rather, phosphate is transferred directly from cytoplasmic enzymes IIB to carbohydrates entering through EIIC transporters. While the PTS is often described as a carbohydrate transport system, it is more aptly conceived as a global regulatory system that senses and responds both to the energetic state of the cell and to environmental nutrient availability.

Among PTS components, glucose-specific EIIA (EIIA^Glc^) is arguably the farthest-reaching regulator of bacterial physiology. In spite of the sugar specificity implied by its name, EIIA^Glc^ activates PTS-dependent transport of many sugars in addition to glucose (2, 3). When these sugars are present and actively transported, EIIA^Glc^ exists predominantly in its unphosphorylated form and can serve as a reporter of the abundance of PTS-dependent sugars.

EIIA^Glc^ interacts directly with both cytoplasmic and membrane-associated proteins to modify their function. For instance, EIIA^Glc^ dephosphorylates HPr, phosphorylates EIIBs, and regulates the function of the cytoplasmic enzymes glycerol kinase and adenylate cyclase (AC). EIIA^Glc^ also interacts directly with several integral membrane proteins to alter their function. Unphosphorylated EIIA^Glc^ binds to and inhibits transporters that import non-PTS sugars in a process known as inducer exclusion (4). Vibrio cholerae EIIA^Glc^ also interacts with the integral membrane protein MshH, which is a homolog of Escherichia coli CsrD (5).

Through *in silico* analysis, we identified an amphipathic α-helix (AH) at the N terminus of V. cholerae EIIA^Glc^. We questioned whether this AH might be critical for EIIA^Glc^ partner regulation. Here, we demonstrate that the N terminus of V. cholerae EIIA^Glc^ mediates membrane association independently of the remainder of the EIIA^Glc^ protein *in vivo*. Our data show that helix-less EIIA^Glc^ completes the PTS phosphotransfer cascade but does not activate PTS-dependent sugar transport. This demonstrates that phosphotransfer can be uncoupled from PTS-dependent transport. We hypothesize that a second interaction at the membrane, possibly with EIIC, is required for EIIA^Glc^ catalysis of PTS-dependent sugar transport.

Functional assays demonstrate that the V. cholerae EIIA^Glc^ AH is essential for regulation of its integral membrane protein partners but dispensable for regulation of cytoplasmic partners. In cells expressing helix-less EIIA^Glc^, the action of cytoplasmic partners is unopposed, resulting in outlying phenotypes in both biofilm formation and metabolism within the mammalian intestine. Thus, we describe a novel role for a bacterial AH in integration of metabolic signals arriving from the cytoplasm with nutritional signals present in the external environment and provide evidence that this function is at work in the mammalian intestine and in the biofilm, a structure associated with arthropod hosts and the environment (6, 7). We propose that this regulation allows cells to fine-tune their physiology and behavior to existing energetic and nutritional constraints and may provide a conserved target for manipulation of pathogen metabolism in the human intestine and the environment.

## RESULTS

### The N-terminal amphipathic helix of V. cholerae EIIA^Glc^ mediates association with the cell membrane *in vivo*.

**FIG 1:**
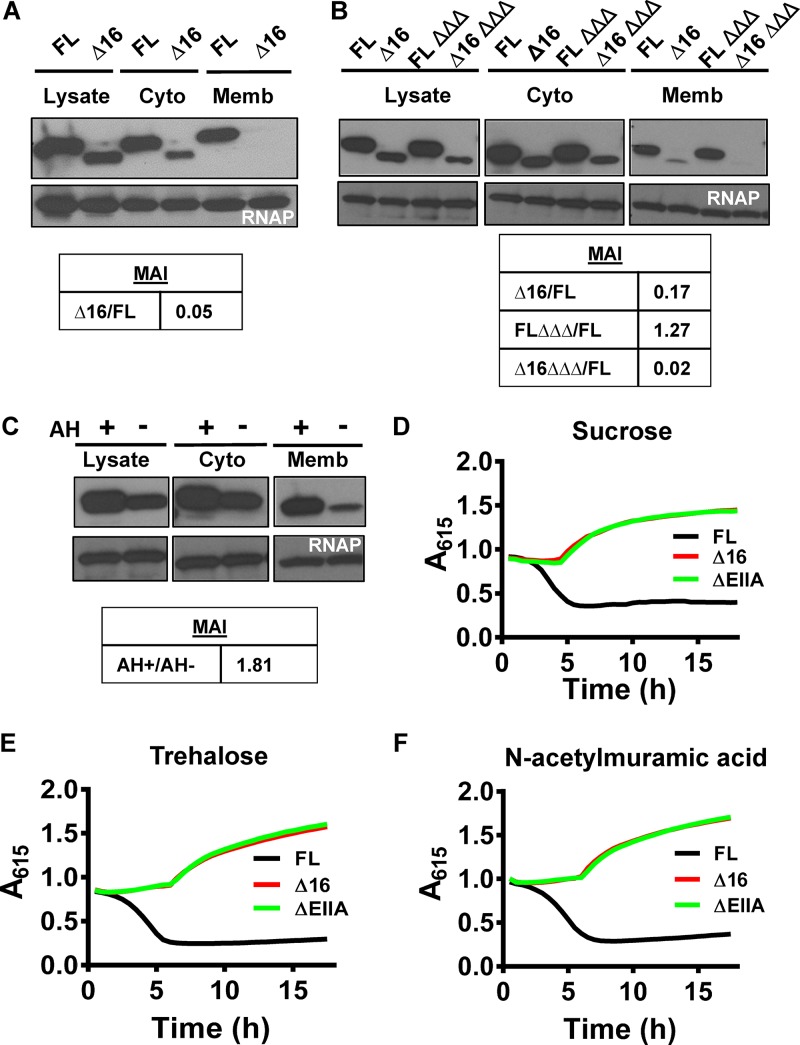
The N terminus of EIIA^Glc^ forms an AH that mediates membrane association and is essential for PTS-dependent activation of sugar transport through EIICs. (A and B) Immunoblots of the lysate, cytoplasmic, and membrane fractions of control or ΔEIIBC^Glc^ Δ*mshH* Δ*cya* mutant (ΔΔΔ) *V. cholerae* cells expressing affinity-tagged full-length EIIA^Glc^ (FL) or Δ16 EIIA^Glc^ (Δ16). The membrane association index (MAI) with respect to FL is given below. (C) Immunoblots of the lysate, cytoplasmic, and membrane fractions of wild-type *V. cholerae* expressing neon green alone (AH−) or as a C-terminal fusion to the AH of EIIA^Glc^ (AH+) encoded on a plasmid. The membrane association index (MAI) with respect to AH− is given below. (D to F) *A*_615_ measured over time for *V. cholerae* expressing the indicated EIIA^Glc^ alleles or deletion (ΔEIIA) and cultured in minimal medium supplemented with pH indicators and sucrose (D), trehalose (E), and *N*-acetylmuramic acid (F). The decrease in *A*_615_ indicates medium acidification due to transport and fermentation of the indicated sugar. Results are representative of three independent experiments.

We previously identified several integral membrane protein partners of V. cholerae EIIA^Glc^ (5) and questioned whether EIIA^Glc^ might interact with the inner membrane. For ease of detection, we included a V5-6×-His affinity tag at the C terminus of full-length EIIA^Glc^ (FL EIIA^Glc^). To determine if FL EIIA^Glc^ was membrane associated, we isolated membrane and cytoplasmic fractions of cells carrying FL EIIA^Glc^ and assessed protein abundance in each fraction. As shown in Fig. 1A, a sizable subpopulation of FL EIIA^Glc^ was present in the membrane fraction. Escherichia coli EIIA^Glc^ has an N-terminal AH that interacts with membranes (8). To ascertain whether this might be the case for V. cholerae EIIA^Glc^, we constructed a helical wheel projection of the N terminus of V. cholerae EIIA^Glc^ using HeliQuest (9). This suggested that the first 16 amino acids of V. cholerae EIIA^Glc^ also form an AH (see Fig. S1 in the supplemental material). The hydrophobic moment of this AH is much larger than that of the N termini of other EIIA homologs encoded in the V. cholerae genome (Fig. S1). To determine whether the EIIA^Glc^ AH was responsible for membrane association, we constructed a mutant encoding an EIIA^Glc^ allele in which the first 16 amino acids comprising the AH were deleted and a V5-6×-His affinity tag was included at the C terminus (Δ16 EIIA^Glc^). Western blot analysis exploiting the affinity tag showed that Δ16 EIIA^Glc^ was less abundant in cells than FL EIIA^Glc^ (Fig. S2A). Cellular fractionations carried out with cells expressing Δ16 EIIA^Glc^ showed that this truncated protein was approximately 20-fold less abundant in the membrane fraction than FL EIIA^Glc^ (Fig. 1A). This suggests that the N terminus of EIIA^Glc^ plays a role in membrane association.

10.1128/mBio.00858-18.1 FIG S1  The hydrophobic moment of the N terminus of EIIA^Glc^ is larger than that of other solo EIIA or EIIABC homologs of V. cholerae. Helical wheel projections of the first 16 amino acids of all V. cholerae EIIA homologs that are either alone or found at the N terminus of a protein. Hydrophobic residues are shown in yellow. The central arrows indicate the magnitude and direction of the hydrophobic moment. The PTS enzyme II domains found in each protein are indicated in parentheses, and the hydrophobic moment (µH) is shown below. Projections were generated with the HeliQuest software. Download FIG S1, PDF file, 0.9 MB. Copyright © 2018 Vijayakumar et al. 2018 Vijayakumar et al. This content is distributed under the terms of the Creative Commons Attribution 4.0 International license.

10.1128/mBio.00858-18.2 FIG S2  Expression, membrane association, and function of mutant EIIA^Glc^ alleles. (A) Western blot analysis of affinity-tagged full-length EIIA^Glc^ (FL) and mutants with the first 16 amino acids removed (Δ16), one (1X-MreB) or two MreB (2X-MreB) amphipathic helices at the N terminus, a K6P point mutant, and one MinD amphipathic helix at the C terminus (MinD). (B) Immunoblots of the lysate (L), cytoplasmic (C), and membrane (M) fractions of V. cholerae strains carrying the indicated EIIA^Glc^ alleles. The membrane association index (MAI) is given below. (C and D) Transport of sucrose (C) and glucose (D) by V. cholerae with a wild-type (WT), C-terminally tagged (FL), or deleted (ΔEIIA) EIIA^Glc^ allele. (E) Transport of glucose by wild-type V. cholerae (WT) and a mutant with deletions of EI, HPr, and EIIA^Glc^ (ΔPTS) alone, carrying a control vector with inducible expression of β-galactosidase (pBAD-lacZ), or carrying a vector with inducible expression of EI (pBAD-EI). Download FIG S2, PDF file, 0.2 MB. Copyright © 2018 Vijayakumar et al. 2018 Vijayakumar et al. This content is distributed under the terms of the Creative Commons Attribution 4.0 International license.

We reasoned that the AH of EIIA^Glc^ might interact with the membrane directly or might promote interactions with partners that are membrane associated. In the latter case, we predicted that we would observe a decrease in membrane association when partners of FL EIIA^Glc^ were removed. To test this, we performed fractionation experiments in a genetic background in which MshH, EIIBC^Glc^, and AC were absent (Fig. 1B). Interestingly, deletion of these three partners of EIIA^Glc^ did not decrease membrane association of FL EIIA^Glc^ but did eliminate most of the residual association of Δ16 EIIA^Glc^ with the membrane. These observations demonstrate that FL EIIA^Glc^ associates with the membrane in the absence of several major partners and also suggest that EIIA^Glc^ interacts with membrane-associated protein partners, albeit weakly, in the absence of its AH.

As a final test of EIIA^Glc^ AH membrane association, we examined the subcellular location of the AH in the absence of the remainder of the protein. To do so, we isolated the cytoplasmic and membrane fractions of V. cholerae strains carrying plasmids expressing the fluorescent protein neon green either alone or fused to the AH of EIIA^Glc^ and compared the relative abundances of the two proteins in the membrane fraction. As shown in Fig. 1C, neon green fused to the EIIA^Glc^ AH was approximately 1.8-fold more abundant in the membrane fraction than neon green alone. This demonstrates that the EIIA^Glc^ AH mediates membrane association independently of the remainder of the EIIA^Glc^ protein.

Because phosphorylation is required for many of the functions of EIIA^Glc^ and has been found to regulate association of other AHs with the cell membrane (10), we questioned whether EIIA^Glc^ membrane association might be regulated by phosphorylation. However, our data showed that substituting an alanine for the phosphorylated histidine at position 91 in FL EIIA^Glc^ and Δ16 EIIA^Glc^ alleles did not alter membrane association (Fig. S2B). Taken together, these results demonstrate that the EIIA^Glc^ AH mediates membrane association and, furthermore, that membrane association is not regulated by the phosphorylation state of EIIA^Glc^.

### The EIIA^Glc^ AH is essential for phosphorylation-dependent sugar entry through EIIC components.

Because it mediates membrane association, we hypothesized that the EIIA^Glc^ AH might selectively alter interactions with integral membrane protein partners. One of the best-studied functions of EIIA^Glc^ is facilitation of PTS-dependent sugar transport, which involves both cytoplasmic and membrane-associated proteins. We reasoned that a study of PTS phosphotransfer and sugar transport in the Δ16 EIIA^Glc^ mutant might yield insights into the function of the EIIA^Glc^ AH. This process depends on EIIA^Glc^ for transfer of phosphate from the cytoplasmic protein HPr to EIIB domains that are cytoplasmic but often fused to EIICs located in the inner membrane.

To determine whether a Δ16 EIIA^Glc^ mutant was competent to carry out sugar transport, we utilized a previously developed assay that depends on medium acidification resulting from fermentation of transported carbohydrates (11). We first measured the impact of the affinity tag on FL EIIA^Glc^ function and found that transport and catabolism of glucose and sucrose were comparable in strains carrying native or tagged EIIA^Glc^ (Fig. S2C and D). Several EIIBC proteins encoded in the V. cholerae genome are not accompanied by a dedicated sugar-specific EIIA, and many of these depend on EIIA^Glc^ for PTS-dependent transport (12, 13). We tested the ability of Δ16 EIIA^Glc^ to mediate transport of several such sugars, including sucrose, trehalose, and *N*-acetylmuramic acid. In each of these cases, Δ16 EIIA^Glc^ and ΔEIIA^Glc^ mutants behaved similarly (Fig. 1D to F). These observations support a role for the AH of EIIA^Glc^ in PTS-dependent sugar transport.

### The AH of EIIA^Glc^ is not essential for phosphotransfer through the PTS.

**FIG 2:**
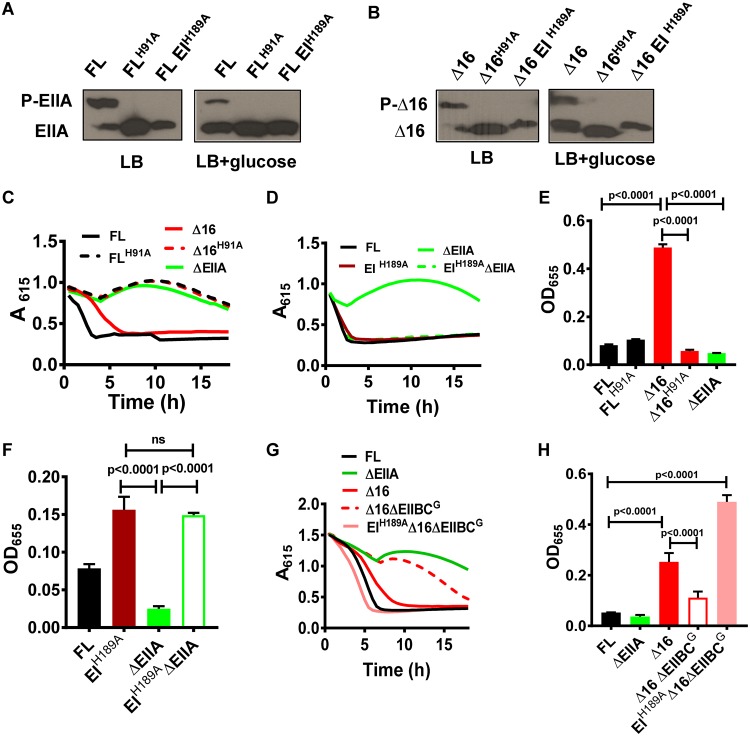
Repression of alternative glucose transport and biofilm formation is mediated by phospho-EI and relieved by EIIA^Glc^ AH-independent phosphotransfer to EIIB. (A and B) Phos-tag acrylamide gel electrophoresis and anti-V5 antibody immunoblotting of the affinity-tagged full-length EIIA^Glc^ (FL), full-length EIIA^Glc^ with histidine 91 mutated to alanine (FL^H91A^), Δ16 EIIA^Glc^ (Δ16), Δ16 EIIA^Glc^ with histidine 91 mutated to alanine (Δ16^H91A^), or EIIA^Glc^ in a strain with histidine 189 of EI mutated to alanine (EI^H189A^) extracted from cells grown in LB alone or supplemented with glucose. (C and D) *A*_615_ measured over time for V. cholerae expressing the indicated EIIA^Glc^ alleles or deletion (ΔEIIA) and cultured in minimal medium supplemented with pH indicators and glucose. (E and F) Quantification of biofilm formation by the indicated V. cholerae strains cultured in MM supplemented with glucose. Data represent the means from three biological replicates, and error bars represent the standard deviation. Statistical significance was calculated using a one-way analysis of variance followed by Tukey’s multiple-comparison test. ns, not significant. (G) *A*_615_ measured over time in cultures of the indicated V. cholerae control or mutant strains. Minimal medium supplemented with pH indicators and glucose was used. Results are representative of three independent experiments. (H) Quantification of biofilm formation by the indicated V. cholerae strains cultured in MM supplemented with glucose. Data represent the means from three biological replicates, and error bars represent the standard deviation. Statistical significance was calculated using a one-way analysis of variance followed by Tukey’s multiple-comparison test.

Phosphotransfer through the PTS is a prerequisite for PTS-dependent sugar transport (14). Because a Δ16 EIIA^Glc^ mutant is defective in PTS-dependent sugar transport, we evaluated the possibility that removal of the EIIA^Glc^ AH might interrupt phosphotransfer through the PTS using Phos-tag acrylamide, which resolves the phosphorylated and unphosphorylated forms of EIIA^Glc^. In LB broth, sizable populations of both native FL EIIA^Glc^ and Δ16 EIIA^Glc^ were in the phosphorylated state (Fig. 2A and B). This demonstrates that the AH is not required for EIIA^Glc^ phosphorylation by HPr. As negative controls, we also tested strains in which the phosphorylated histidine of EI (EI^H189A^) or EIIA^Glc^ (EIIA^GlcH91A^) was mutated to alanine. In these strains, the phosphorylated form of EIIA^Glc^ was not observed (Fig. 2A and B). Furthermore, when glucose was added to LB, an increased amount of both FL EIIA^Glc^ and Δ16 EIIA^Glc^ was observed in the unphosphorylated state. This suggests that Δ16 EIIA^Glc^ passes phosphate to downstream components in the presence of glucose and, therefore, that the AH of EIIA^Glc^ is not essential for phosphotransfer through the PTS. Although Δ16 EIIA^Glc^ completes the PTS phosphotransport cascade, it is unable to transport sugar via the PTS. We conclude that removal of the EIIA^Glc^ AH uncouples phosphotransfer from PTS-dependent carbohydrate transport. Furthermore, our observations point to an additional AH-dependent role for EIIA^Glc^ in activating sugar transport.

### Dephosphorylation of EI by EIIA^Glc^ activates alternative glucose transport.

V. cholerae transports and phosphorylates glucose via the hybrid PTS protein EIIBC^Glc^. This transport depends on EIIA^Glc^ for phosphotransfer through the PTS. V. cholerae also expresses an alternative glucose transporter that does not phosphorylate glucose during transport (13, 15). This alternative glucose transporter is also active only in the presence of EIIA^Glc^ but does not require the upstream PTS components EI and HPr (2, 15). Because EIIA^Glc^ is essential for both PTS-dependent and alternative glucose transport, a ΔEIIA^Glc^ mutant does not transport glucose. By extrapolation from our finding that Δ16 EIIA^Glc^ is unable to mediate PTS-dependent transport of sucrose, trehalose, and *N*-acetylmuramic acid, we reasoned that PTS-dependent transport of glucose should be defective as well. To determine whether alternative transport was active in a Δ16 EIIA^Glc^ mutant, we measured glucose fermentation by this mutant. In fact, we found that the Δ16 EIIA^Glc^ mutant does transport glucose, albeit less efficiently than a strain encoding FL EIIA^Glc^ (Fig. 2C). We attribute this decrease in efficiency to the absence of PTS-dependent transport and conclude that the AH is not essential for EIIA^Glc^ activation of alternative glucose transport.

As we had already established that Δ16 EIIA^Glc^ participates in the PTS phosphotransfer cascade, we questioned whether activation of alternative glucose transport by EIIA^Glc^ might be the result of phosphotransfer. To test this, we compared glucose transport by a strain expressing the unphosphorylatable allele Δ16 EIIA^GlcH91A^ with that by a control strain expressing Δ16 EIIA^Glc^ (Fig. 2C). Our results showed that mutation of the Δ16 EIIA^Glc^ phosphorylation site prevents glucose transport. We reasoned that this could indicate either direct activation of transport by phospho-EIIA^Glc^ or inhibition of transport by phospho-EI, which would be predicted to accumulate in a Δ16 EIIA^GlcH91A^ mutant. To distinguish between these possibilities, we constructed a strain carrying an EI allele that mimics unphosphorylated EI (EI^H189A^) in a ΔEIIA^Glc^ mutant background and tested its ability to transport glucose. This EI^H189A^ mutant did, in fact, restore alternative glucose transport to the ΔEIIA^Glc^ mutant (Fig. 2D). Furthermore, in a ΔPTS mutant with deletions of EI, HPr, and EIIA^Glc^, plasmid-based expression of EI was sufficient to repress alternative glucose transport (Fig. S2E). Our findings indicate that both EIIA^Glc^ and Δ16 EIIA^Glc^ activate alternative glucose transport through their ability to dephosphorylate EI.

### EIIA^Glc^ regulation of biofilm formation is the sum of opposing AH-dependent and AH-independent regulatory mechanisms.

Some partners of V. cholerae EIIA^Glc^ activate biofilm formation, while others repress it (5, 12). We hypothesized that a helix-less EIIA^Glc^ mutant might reveal a biofilm phenotype intermediate between that of strains expressing FL EIIA^Glc^ or no EIIA^Glc^ due to its ability to regulate only a subset of EIIA^Glc^ partners. In fact, a Δ16 EIIA^Glc^ mutant demonstrated a greatly increased propensity for surface association compared with both the control strain encoding FL EIIA^Glc^ and the ΔEIIA^Glc^ mutant (Fig. 2E) (15).

We previously reported a regulatory role for phospho-EI in repression of V. cholerae biofilm formation (15). Therefore, we mutated the phosphorylation site of Δ16 EIIA^Glc^ to determine if this might reduce AH-independent activation of biofilm formation. In fact, this decreased biofilm formation to levels similar to that of a ΔEIIA^Glc^ mutant (Fig. 2E). Conversely, addition of an EI^H189A^ allele in a ΔEIIA^Glc^ mutant background restored biofilm formation (Fig. 2F). We conclude that, similarly to alternative glucose transport, Δ16 EIIA^Glc^ activates biofilm formation by decreasing the intracellular concentration of phospho-EI. Based on these results, we propose that AH-dependent and AH-independent partners of FL EIIA^Glc^ have opposing impacts on biofilm formation, that removal of the AH results in unopposed activation of biofilm formation, and that synthesis of these opposing regulatory forces depends on the presence of the EIIA^Glc^ AH.

### Helix-less EIIA^Glc^ is competent to complete the PTS phosphotransfer cascade by passing phosphate to EIIBC^Glc^.

Our phosphorylation studies suggested that Δ16 EIIA^Glc^ receives phosphate from HPr and passes it to unidentified downstream components. While EIIB^Glc^ is a cytoplasmic domain, it is predicted to be close to the membrane due to its fusion to EIIC^Glc^. Therefore, the ability of Δ16 EIIA^Glc^ to transfer phosphate to EIIBC proteins would suggest that the AH is not required for phosphotransfer to membrane-associated components and, furthermore, that phosphotransfer is not sufficient to catalyze PTS-dependent sugar transport. We hypothesized that, if Δ16 EIIA^Glc^ passed phosphate to the EIIB moiety of EIIBC^Glc^, deletion of EIIBC^Glc^ in a Δ16 EIIA^Glc^ mutant background would increase the concentration of phospho-EI, leading to repression of alternative glucose transport and biofilm formation. In fact, we observed inhibition of alternative glucose transport and biofilm formation in a Δ16 EIIA^Glc^ ΔEIIBC^Glc^ double mutant (Fig. 2G and H). Furthermore, incorporation of an EI^H189A^ allele restored both glucose transport and biofilm formation to the Δ16 EIIA^Glc^ ΔEIIBC^Glc^ mutant (Fig. 2G and H). These studies provide further evidence that helix-less EIIA^Glc^ not only receives phosphate from HPr but also transfers it to EIIBC^Glc^. Although Δ16 EIIA^Glc^ is competent to complete the PTS phosphotransfer cascade, it cannot activate PTS-dependent sugar transport. We hypothesize that an additional interaction at the membrane, possibly with EIIC, is required for facilitation of transport by EIIA^Glc^.

### The EIIA^Glc^ AH is required for activation of additional integral membrane protein partners.

We showed that the AH of EIIA^Glc^ was dispensable for phosphotransfer, which is carried out by cytoplasmic proteins. In contrast, this AH was essential for PTS-dependent sugar transport, which involves the integral membrane EIIC protein. We hypothesized that the AH might be required specifically for interactions with integral membrane protein partners of EIIA^Glc^, and therefore, we tested the role of the AH in regulation of two additional integral membrane protein partners of EIIA^Glc^, the maltose transporter and MshH.

**FIG 3:**
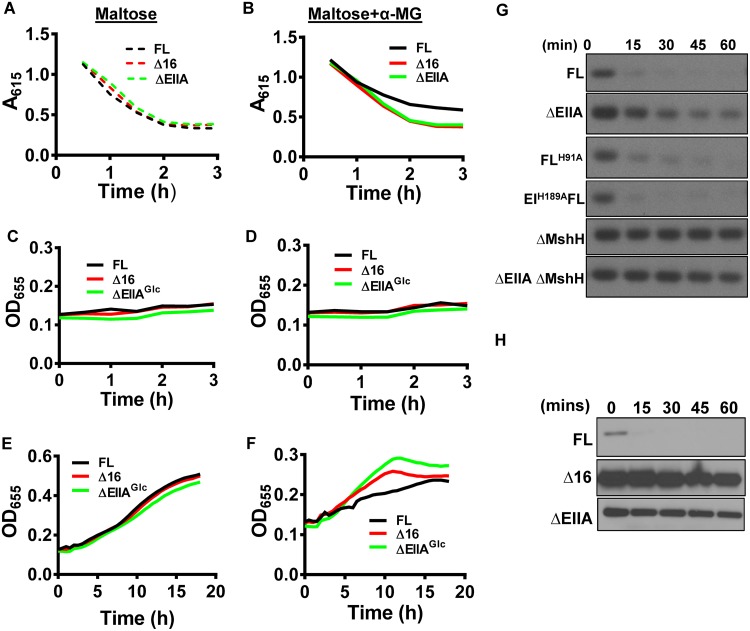
EIIA^Glc^ regulation of integral membrane protein partners is AH dependent. (A to F) Medium acidification (A and B) and growth curves (C to F) carried out in minimal medium supplemented with pH indicators and maltose alone (maltose) or supplemented with α-methylglucoside (maltose + α-MG). (G and H) Northern blot assay of the sRNA *csrB* in LB-cultured, mid-log-phase cells of the indicated genotype. Samples were harvested at the noted times after addition of rifampin to arrest transcript elongation.

When glucose is plentiful, unphosphorylated E. coli EIIA^Glc^ binds to MalK, a component of the maltose-specific ABC transporter, to inhibit maltose entry into the cell (16, 17). This process, known as inducer exclusion, prevents maltose from inducing transcription of genes required for its transport and catabolism (4, 18). To test whether this regulatory process is dependent on the EIIA^Glc^ AH in V. cholerae, we assessed fermentation of maltose in the presence of the glucose analog α-methyl glucoside (α-MG). α-MG is transported and phosphorylated, resulting in dephosphorylation of EIIA^Glc^. Because α-MG cannot be fermented, medium acidification reflects maltose transport and fermentation (19). While FL EIIA^Glc^ did not modulate maltose fermentation in the absence of α-MG (Fig. 3A), maltose catabolism by a strain encoding FL EIIA^Glc^ was less in the presence of this glucose analog (Fig. 3B). In contrast, fermentation of maltose by the V. cholerae Δ16 EIIA^Glc^ and ΔEIIA^Glc^ mutants in the presence of α-MG were comparable. Although growth rates of these strains were similar over the course of fermentation experiments (Fig. 3C and D), at later time points, growth of the Δ16 EIIA^Glc^ and ΔEIIA^Glc^ mutants was greater than that of wild-type V. cholerae (Fig. 3E and F), likely due to differences in maltose utilization. This demonstrates that the AH of V. cholerae EIIA^Glc^ is required for maltose exclusion.

CsrA, a key component of the carbon storage regulatory system, is a small RNA-binding protein that regulates the abundance of many proteins through effects on RNA stability and translation in both V. cholerae and E. coli (20, 21). CsrA activity is inhibited by the small *csr* RNAs (22). In conjunction with RNase E, E. coli CsrD degrades the *csr* RNAs in an EIIA^Glc^-dependent fashion (23, 24). The V. cholerae homolog of E. coli CsrD is MshH, originally named for its position in the operon encoding the mannose-sensitive hemagglutinin (25). In spite of its homology with E. coli CsrD, this name was retained because the third V. cholerae *csr* small RNA (sRNA) had already been given the name *csrD* (5, 26). EIIA^Glc^ activates degradation of the *csr* sRNAs through a direct interaction with MshH (5, 24). In E. coli, unphosphorylated EIIA^Glc^ activates *csrB* degradation, and degradation can be restored in a ΔEIIA^Glc^ mutant by provision of an EIIA^GlcH91A^ allele in *trans* (24). Similarly, we found that both EIIA^GlcH91A^ and an EI^H189A^ point mutant, which cannot transfer phosphate through HPr to EIIA^Glc^, activated degradation of *csrB* (Fig. 3C). To determine whether the EIIA^Glc^ AH was essential for *csrB* degradation, we measured degradation of *csrB* over time in V. cholerae expressing FL EIIA^Glc^, Δ16 EIIA^Glc^, or no EIIA^Glc^ (Fig. 3D). *csrB* degradation was observed only in strains expressing FL EIIA^Glc^. This demonstrates that EIIA^Glc^ activates the integral membrane protein MshH by an AH-dependent mechanism. Based on our observations, we propose that the AH of EIIA^Glc^ is specifically required for activation of integral membrane protein partners.

### EIIA^Glc^ regulation of integral membrane protein partners does not depend on AH sequence.

**FIG 4:**
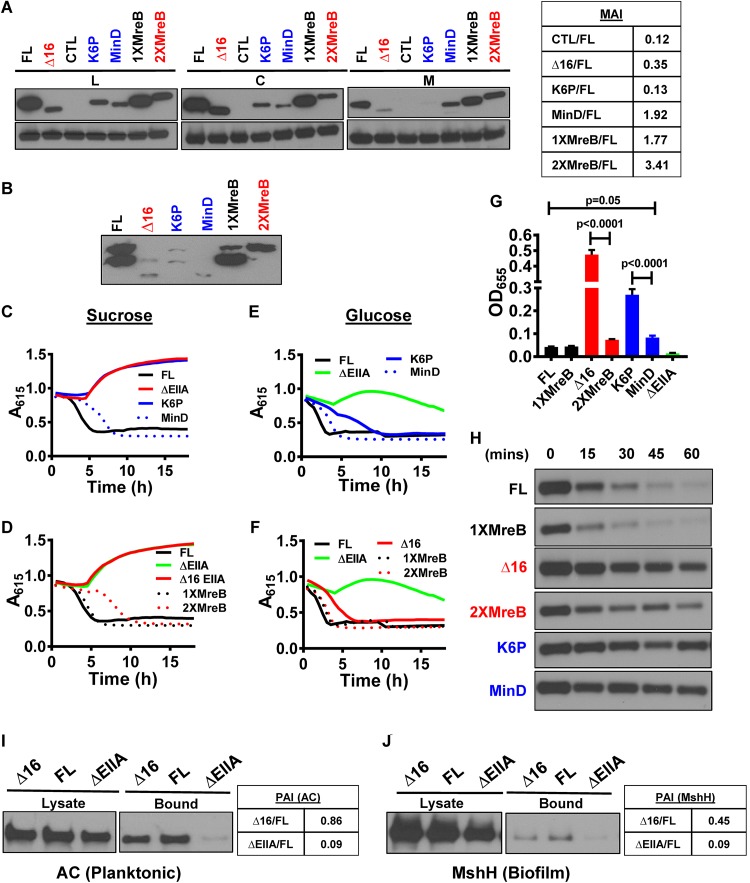
AH swapping establishes that the function of the V. cholerae EIIA^Glc^ AH is not amino acid sequence dependent. (A) Anti-V5 antibody immunoblot assays of the lysate (L), cytoplasmic (C), and membrane (M) fractions of V. cholerae expressing the indicated affinity-tagged EIIA^Glc^ constructs. The membrane association index (MAI) with respect to FL is given at the right. (B) Phos-tag acrylamide gel electrophoresis and anti-V5 antibody immunoblot assay of V. cholerae expressing the indicated EIIA^Glc^ alleles. *A*_615_ measured over time in cultures of V. cholerae expressing FL-EIIA^Glc^ (FL), K6P-EIIA^Glc^ (K6P), 1X-MreB EIIA^Glc^ (1XMreB), 2X-MreB EIIA^Glc^ (2XMreB), EIIA^Glc^-MinD (MinD), and ΔEIIA^Glc^ (ΔEIIA). (C to F) Minimal medium supplemented with pH indicators and sucrose (C and D) or glucose (E and F) was used. Results are representative of three independent experiments. (G) Quantification of biofilm formation by strains as indicated in minimal medium supplemented with glucose. Bars represent the mean from biological triplicates. Error bars indicate the standard deviation. Statistical significance was calculated using a one-way analysis of variance followed by Tukey’s multiple-comparison test. (H) Northern blot of the sRNA *csrB* in LB-cultured, mid-log-phase V. cholerae cells that express the indicated EIIA^Glc^ alleles. Samples were harvested at the noted times after addition of rifampin to arrest transcription elongation. (I and J) Immunoblots of adenylate cyclase (AC) (I) and MshH (J) in cell lysates and the bound fractions that coimmunoprecipitated with FL EIIA^Glc^ or Δ16 EIIA^Glc^. The partner association index (PAI) with respect to FL is given at the right.

We questioned whether the EIIA^Glc^ AH interacted with integral membrane protein partners in a sequence-specific manner or whether it simply stabilized this interaction through membrane association. The E. coli protein MreB and the B. subtilis protein MinD harbor AHs at their N and C termini, respectively, which anchor these proteins to the cell membrane (27, 28). To evaluate the dependence of EIIA^Glc^ AH function on amino acid sequence, we swapped the EIIA^Glc^ AH with one (1X-MreB EIIA^Glc^) or two (2X-MreB EIIA^Glc^) copies of the MreB AH and also fused the MinD AH to the C terminus of Δ16 EIIA^Glc^ (EIIA^Glc^-MinD) (Fig. S3A). As a negative control, we constructed a chromosomal point mutant in which the lysine residue at position 6 of the EIIA^Glc^ AH was replaced with proline to disrupt the AH (K6P-EIIA^Glc^). The abundances of these mutant alleles in cells were quite different (Fig. S2A). To account for this, we compared the phenotypes of alleles with similar expression patterns. For example, FL EIIA^Glc^ was compared to 1X-MreB EIIA^Glc^, Δ16 EIIA^Glc^ was compared to 2X-MreB EIIA^Glc^, and K6P-EIIAGlc was compared to EIIA^Glc^-MinD. We first tested membrane association of these fusion proteins. As predicted, K6P-EIIA^Glc^ demonstrated very little membrane association (Fig. 4A). Addition of one MreB AH to Δ16 EIIA^Glc^ restored membrane association, and as has previously been noted (28), a second MreB AH increased it even more. Addition of the AH of MinD to the C terminus of Δ16 EIIA^Glc^ also resulted in membrane association. We conclude that other bacterial AHs can mediate EIIA^Glc^ membrane association.

10.1128/mBio.00858-18.3 FIG S3  Maltose exclusion does not depend on AH sequence. (A) Amino acid sequences of wild-type and alternative amphipathic helices combined with Δ16-EIIA^Glc^. (B) Absorbance at 615 nm over time in minimal medium supplemented with maltose and α-methylglucoside is shown for strains encoding affinity-tagged full-length EIIA^Glc^ (FL) and Δ16 EIIA^Glc^ (Δ16), 1X-MreB EIIA^Glc^ (1X MreB) or 2X-MreB EIIA^Glc^ (2X MreB), K6P-EIIA^Glc^ (K6P), EIIA^Glc^-MinD (MinD), and ΔEIIA^Glc^ mutants. Download FIG S3, PDF file, 0.1 MB. Copyright © 2018 Vijayakumar et al. 2018 Vijayakumar et al. This content is distributed under the terms of the Creative Commons Attribution 4.0 International license.

As a control for activity, we confirmed that both phosphorylated and unphosphorylated forms of EIIA^Glc^, Δ16 EIIA^Glc^, and K6P-EIIA^Glc^ were present and, therefore, that these proteins could participate in phosphotransfer (Fig. 4B). However, increasing membrane association was correlated with a decrease in the abundance of the phosphorylated forms of these proteins. One possibility is that strong association with the cell membrane disfavors phosphorylation by cytoplasmic PTS components.

We then assessed sucrose transport, which depends on the AH of EIIA^Glc^. As expected, the strain carrying the K6P allele, which does not associate with the cell membrane, showed no evidence of sucrose fermentation (Fig. 4C). Strains carrying EIIA^Glc^ alleles with heterologous AHs fermented sucrose, consistent with sucrose transport through the PTS (Fig. 4C and D). Similarly, glucose fermentation curves were similar to that of a strain expressing FL EIIA^Glc^ (Fig. 4E and F). Our results demonstrate that activation of EIIC sugar transport by EIIA^Glc^ depends neither on the amino acid sequence nor the position of its amphipathic helix.

We questioned whether the EIIA^Glc^ alleles carrying heterologous AHs could mediate maltose exclusion. We found that the 1X-MreB and, to some extent, the 2X-MreB EIIA^Glc^ alleles inhibited maltose transport (Fig. S3B). In contrast, the MinD and K6P-EIIA^Glc^ alleles did not. These results demonstrate that the role of the EIIA^Glc^ AH in maltose exclusion again does not depend on amino acid sequence. However, we cannot rule out a requirement for an N-terminal position.

We have demonstrated opposing roles for FL EIIA^Glc^ and helix-less EIIA^Glc^ in regulation of biofilm formation. Because the heterologous AHs other than K6P restored biofilm formation to the levels of strains encoding FL EIIA^Glc^, we conclude that integration of these opposing regulatory forces does not depend on AH sequence (Fig. 4G). Similarly, with the exception of the MinD AH, these heterologous AHs activated MshH (Fig. 4H) (5). Taken together, our data suggest that interaction with the cell membrane is the principal role of the AH in EIIA^Glc^ regulation of integral membrane protein partners.

### The N termini of diverse EIIA^Glc^ homologs are poorly conserved but retain amphipathicity.

If the EIIA^Glc^ AH simply functioned as a membrane association domain, one might predict that the evolutionary pressure to conserve the primary amino acid sequence of the AH would be less than that of the protein core. To evaluate this, we aligned the sequences of EIIA^Glc^ homologs found in several distantly related bacterial species (Fig. S4). Indeed, the N-terminal sequences of these proteins were divergent, and an amino acid position beyond which the sequences were highly conserved was identified. In spite of poor sequence conservation, the N termini of the EIIA^Glc^ homologs all retained their amphipathic nature with the exception of those of *Streptomyces* and Burkholderia pseudomallei (Fig. S5). We hypothesize that the EIIA^Glc^ homologs from these two species either do not regulate integral membrane protein partners or associate with the membrane by an alternative mechanism. Our findings are consistent with a conserved sequence-independent function for the EIIA^Glc^ AH.

10.1128/mBio.00858-18.4 FIG S4  The sequence of the EIIA^Glc^ AH is not conserved. Clustal Omega was used to align the sequences of the EIIA^Glc^ homologs of Borrelia burgdorferi, *Streptomyces* ScaeMP-e83, Streptococcus pneumoniae, Vibrio cholerae, Escherichia coli, Burkholderia pseudomallei, Staphylococcus aureus, and Listeria monocytogenes. Red font is used to highlight the amino acid separating the poorly conserved N terminus of EIIA^Glc^ from the protein core. While the sequence of the body of EIIA^Glc^ is conserved, the N terminus varies. Download FIG S4, PDF file, 0.1 MB. Copyright © 2018 Vijayakumar et al. 2018 Vijayakumar et al. This content is distributed under the terms of the Creative Commons Attribution 4.0 International license.

10.1128/mBio.00858-18.5 FIG S5  In spite of poor N-terminal amino acid sequence conservation, the EIIA^Glc^ proteins of unrelated bacteria have a predicted N-terminal amphipathic helix. HeliQuest software was used to identify amphipathic helices in the poorly conserved N termini of EIIA^Glc^ proteins from the indicated organisms. In each case except that of the distantly related *Streptomyces* and *Burkholderia* species, an 11-amino-acid amphipathic helix, whose sequence is listed below the bacterial name, was found at the very beginning of the protein. The hydrophobic moment µH for each helix is given below. Download FIG S5, PDF file, 0.8 MB. Copyright © 2018 Vijayakumar et al. 2018 Vijayakumar et al. This content is distributed under the terms of the Creative Commons Attribution 4.0 International license.

### The N-terminal AH stabilizes V. cholerae EIIA^Glc^ interactions with an integral membrane protein partner but not a cytoplasmic one.

To directly probe the role of membrane association in EIIA^Glc^ protein partner recognition, we assessed coimmunoprecipitation of AC, a cytoplasmic partner, and MshH, an integral membrane protein partner, with the FL and Δ16 EIIA^Glc^ alleles. AC principally associates with EIIA^Glc^ in the planktonic state, while MshH associates with EIIA^Glc^ in the biofilm state (5). Therefore, we harvested cells in the planktonic and biofilm states to detect interactions with AC and MshH, respectively. Comparable amounts of AC coimmunoprecipitated with FL and Δ16 EIIA^Glc^ (Fig. 4I). In contrast, approximately half as much MshH coimmunoprecipitated with Δ16 EIIA^Glc^ as with FL EIIA^Glc^ (Fig. 4J). This suggests that, while the AH is not required for recognition of MshH by EIIA^Glc^, it enhances this partner interaction. We propose that AH of EIIA^Glc^ serves as a membrane anchor that stabilizes its interactions with integral membrane partners.

### Cytoplasmic and integral membrane protein partners of EIIA^Glc^ modulate V. cholerae metabolism in the mammalian intestine.

**FIG 5:**
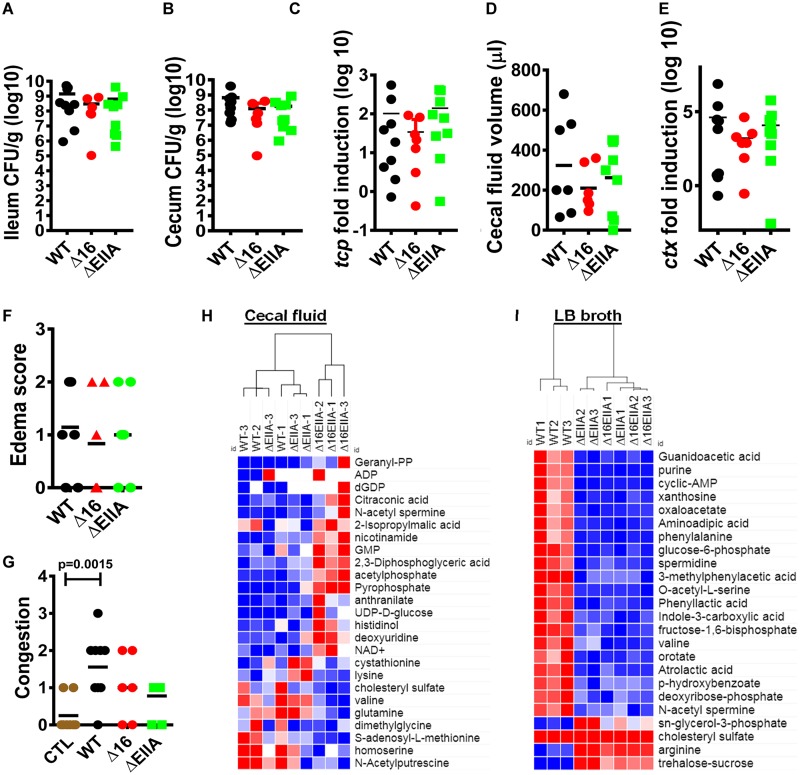
The metabolic environments created by V. cholerae Δ16 EIIA^Glc^ and ΔEIIA^Glc^ strains in cecal fluid are distinct and independent of classical virulence factors. (A and B) Colonization levels of indicated V. cholerae strains in the ileum (A) and colon (B). (C) qRT-PCR quantification of *tcpA* in ileal tissue. (D) Cecal fluid volume. (E) qRT-PCR quantification of *ctxA* in ileal tissue. (F and G) Edema (F) and congestion (G) score according to rubric shown in Data Set S1 and Fig. S4. A one-way analysis of variance followed by Dunnett’s multiple-comparison test was used to calculate statistical significance. (H and I) Cluster analysis of the 25 most significantly different metabolites in the supernatants of cecal fluid harvested from infant rabbits (H) or spent LB broth supernatants (I) inoculated with the indicated V. cholerae strains. Statistical significance was calculated by a one-way analysis of variance followed by a Fisher least significant difference test. Hierarchical clustering was performed using a Euclidean distance algorithm within Morpheus software from the Broad Institute (54). Intensities were mapped to colors using the minimum and maximum of each row independently with red representing values higher than the mean and blue representing values lower than the mean.

A myriad of metabolites derived from intestinal bacteria such as short-chain fatty acids, d-amino acids, and aromatic amino acid derivatives are sensed by the host epithelium and alter intestinal permeability, innate immunity, and host metabolism (29–32). It is, therefore, essential to understand the mechanisms by which diarrheal pathogens alter intestinal metabolites. To determine whether V. cholerae EIIA^Glc^ and its AH might modulate the intestinal environment, we set out to compare the metabolite compositions of the cecal fluid of infant rabbits infected with either wild-type V. cholerae, a Δ16 EIIA^Glc^ mutant, or a ΔEIIA^Glc^ mutant. The toxin-coregulated pilus (TCP) is essential for V. cholerae colonization of the mammalian intestine (33), while cholera toxin (CT) causes the intestinal fluid accumulation associated with cholera (34). Because differences in intestinal colonization or cholera toxin expression would confound the interpretation of our metabolomic analyses, we first established that these classical virulence factors were comparable between the different infections. In fact, we found that colonization of the ileum and cecum by wild-type V. cholerae and the Δ16 EIIA^Glc^ and ΔEIIA^Glc^ mutants did not differ significantly (Fig. 5A and B). Supporting this, no difference was observed in ileal expression of *tcpA*, a gene encoding an essential building block of the toxin-coregulated pilus (Fig. 5C). Substantial amounts of cecal fluid accumulation were observed in the intestines of all colonized animals (Fig. 5D), and we did not measure a significant difference in transcription of *ctxA*, which encodes the A subunit of CT (Fig. 5E). Histological analysis was performed on the terminal ilea of these animals using a standardized grading scale for congestion and edema (Fig. S6). Although a significant difference in congestion was noted only between uninfected animals and those infected with wild-type V. cholerae, no significant difference in intestinal edema or arterial congestion was detected between the three infected animals (Fig. 5F and G). This suggests that *in vivo* expression of the canonical virulence factors, intestinal colonization, and fluid production are not affected by deletion of EIIA^Glc^ or its AH. We concluded, therefore, that metabolic analysis of cecal fluid would not reflect a difference in the intestinal burden or virulence of the V. cholerae strains under study but rather a difference in the metabolisms of these strains in the intestinal environment.

10.1128/mBio.00858-18.6 FIG S6  Scoring rubric for intestinal congestion and edema**:** hematoxylin-and-eosin-stained sections of the infant rabbit terminal ileum harvested at 22 h after V. cholerae infection showing grade 1 and 2 edema and grade 1 to 3 capillary congestion as noted. Pictures were taken at ×20 magnification. Bars, 500 µm (A to E) and 1 mm (F). Download FIG S6, PDF file, 0.5 MB. Copyright © 2018 Vijayakumar et al. 2018 Vijayakumar et al. This content is distributed under the terms of the Creative Commons Attribution 4.0 International license.

To assess the role of cytoplasmic and membrane-associated partners of EIIA^Glc^ in bacterial metabolism within the mammalian intestine, we then performed polar metabolomics on cecal fluid using liquid chromatography-tandem mass spectrometry (LC-MS/MS) (Data Set S1). The specific colonization and virulence parameters of each rabbit used for cecal fluid metabolomic analysis are denoted by a diamond in Fig. S7. The 30 most abundant metabolites identified in cecal fluid harvested from wild-type V. cholerae infection are shown in Fig. S8 and Data Set S1. These include amino acids and related small molecules such as proline, *N*-acetyl-l-alanine, betaine, and creatine and carbohydrates such as succinate and methylmalonic acid.

10.1128/mBio.00858-18.10 DATA SET S1  Metabolomic analysis of infant rabbit cecal fluid and LB supernatants inoculated with wild-type V. cholerae or the indicated EIIA^Glc^ mutant (normalized to sum). Significantly different metabolites were identified using a one-way analysis of variance with a false discovery rate of 0.1 followed by Fisher’s least significant difference test. Download DATA SET S1, XLSX file, 0.1 MB. Copyright © 2018 Vijayakumar et al. 2018 Vijayakumar et al. This content is distributed under the terms of the Creative Commons Attribution 4.0 International license.

10.1128/mBio.00858-18.7 FIG S7  Characteristics of individual infant rabbits from which cecal fluid was harvested for metabolomic analysis. Panels show colonization, cecal fluid accumulation, *ctx* transcription, and *tcpA* transcription for individual infant rabbits. Diamonds denote infant rabbits from which cecal fluid was harvested for metabolomics studies. ND, not done. WT 1 to 5, ΔEIIA 1 and 2, and Δ16 1 and 2 came from litter 1. WT 6 to 9, ΔEIIA 3 to 10, and Δ16 3 to 8 came from litter 2. Download FIG S7, PDF file, 0.2 MB. Copyright © 2018 Vijayakumar et al. 2018 Vijayakumar et al. This content is distributed under the terms of the Creative Commons Attribution 4.0 International license.

10.1128/mBio.00858-18.8 FIG S8  Thirty most abundant metabolites in cecal fluid of infant rabbits infected with wild-type V. cholerae. Download FIG S8, PDF file, 0.04 MB. Copyright © 2018 Vijayakumar et al. 2018 Vijayakumar et al. This content is distributed under the terms of the Creative Commons Attribution 4.0 International license.

The ΔEIIA^Glc^ mutant infection resulted in significant differences in levels of *S*-adenosylmethionine and 2,3-diphosphoglyceric acid compared with wild-type V. cholerae infection, demonstrating that EIIA^Glc^ is active in the intestinal environment (Data Set S1). However, a greater number of significant differences were observed between wild-type V. cholerae and a Δ16 EIIA^Glc^ mutant. In fact, when cluster analysis was performed on the top 25 cecal fluid metabolites ranked according to adjusted *P* value, those from the Δ16 EIIA^Glc^ mutant infection proved to be quite different, while those from the wild-type and ΔEIIA^Glc^ mutant infections clustered more closely together (Fig. 5H). Therefore, similarly to what was observed for biofilm formation, the unopposed action of EIIA^Glc^ cytoplasmic partners in a Δ16 EIIA^Glc^ mutant background resulted in an outlying phenotype. To assess the roles of cytoplasmic and membrane-associated partners of EIIA^Glc^ in a different environment, we performed metabolomic analysis on the supernatants of cells cultured in LB broth (Data Set S1). In this medium, Δ16 EIIA^Glc^ behaved similarly to a ΔEIIA^Glc^ mutant (Fig. 5I). We conclude that, while both cytoplasmic and membrane-associated partners of EIIA^Glc^ participate in creating the metabolic environment of the V. cholerae-infected mammalian intestine, membrane-associated partners of EIIA^Glc^ are dominant in metabolic manipulation of the extracellular environment in LB broth.

## DISCUSSION

V. cholerae EIIA^Glc^, a component of the PTS phosphotransfer cascade, is comprised of an ordered protein core and an N-terminal domain that is predicted to form an AH. Here, we show that this N-terminal domain functions in a sequence-independent manner to mediate EIIA^Glc^ membrane association, a function that is essential for EIIA^Glc^ regulation of membrane-associated but not cytoplasmic protein partners. Because helix-less EIIA^Glc^ transfers phosphate to the cytoplasmic EIIB domain but cannot facilitate sugar transport, we suggest that a direct interaction of EIIA^Glc^ with EIIC at the membrane is also required. This calls into question the long-standing model of PTS-dependent sugar transport in which EIIA^Glc^ participates exclusively as a component of the phosphotransfer cascade. In addition, this investigation of helix-less EIIA^Glc^ reveals, for the first time, the opposing regulatory roles for the cytoplasmic and integral membrane protein partners of EIIA^Glc^ in biofilm formation and metabolism within the mammalian intestine. We propose that these opposing forces are integrated by full-length EIIA^Glc^ in response to internal energetic and external nutritional cues.

The role of the V. cholerae EIIA^Glc^ AH in regulation of integral membrane protein partners may be widely conserved. The N terminus of E. coli EIIA^Glc^ plays a role similar to that of V. cholerae. Structural studies show that 18 amino acids at the N terminus of Escherichia coli EIIA^Glc^ are disordered but form an amphipathic α-helix (AH) in the proximity of phospholipids (8, 35). Furthermore, loss of the N terminus diminishes inducer exclusion and phosphotransfer to incoming sugars but does not block phosphorylation (4, 36). Because our *in silico* studies show that amphipathic sequences are also present at the N terminus of EIIA^Glc^ in many unrelated bacteria, it seems likely that this function of the N terminus is generally conserved in bacteria whose genomes encode the PTS.

Here, we have shown that the presence of the AH allows V. cholerae EIIA^Glc^ to integrate opposing outputs from partners in the cytoplasm and the bacterial inner membrane. We hypothesize that EIIA^Glc^ membrane association is regulated to provide flexibility in responding to cytoplasmic and external signals. There is evidence in the literature for two mechanisms of regulation. Many years ago, investigators determined that a membrane-associated metalloprotease removes the N-terminal AH of EIIA^Glc^ of Salmonella enterica serovar Typhimurium and E. coli (37). However, the role of this cleavage in EIIA^Glc^ function was not elucidated, and we have not observed N-terminal proteolysis of V. cholerae EIIA^Glc^ here. In addition, a recent comprehensive analysis of acetylation sites in the V. cholerae proteome suggests that the lysine at position 6 within the AH is acetylated (38). We predict that acetylation of the EIIA^Glc^ AH should also modulate membrane association.

AHs are found in both prokaryotic and eukaryotic proteins, where they mediate membrane association by inserting into one leaflet of a lipid bilayer (10). The lipid bilayer accommodates this process through altered membrane curvature (39). Thus, AHs serve as both sensors and effectors of cell membrane curvature (40). They may activate membrane-associated proteins that favor high cell curvature, target AH-containing proteins to regions of the membrane that can accommodate this curvature, or nucleate multiprotein complexes at sites of high membrane curvature (10, 40). Such functions are predicted to be sequence independent, and this is, in fact, what we observe for the AH of EIIA^Glc^. While we have noted that the V. cholerae EIIA^Glc^ AH stabilizes interactions with integral membrane protein partners through its association with the membrane, it is possible that additional effects on local membrane structure promote direct partner regulation or catalyze the formation of protein complexes essential for function.

Studies of V. cholerae virulence have focused on the canonical virulence factors TCP and CT, which are associated with host death. However, only a small percentage of cholera cases are lethal. In a *Drosophila melanogaster* model, we have shown that generation of the short-chain fatty acid acetate by commensal bacteria is essential for transcription of a subset of enteroendocrine peptides in the gut that are essential for normal lipid metabolism and development (41). Furthermore, during infection, V. cholerae consumption of acetate and methionine sulfoxide, through the actions of pathogen acetyl coenzyme A (acetyl-CoA) synthase and the glycine cleavage system, respectively, results in host metabolic demise (42, 43). Here, we provide evidence that metabolites that alter the intestinal milieu and epithelial regeneration accumulate in cecal fluid during V. cholerae infection. These include creatine, which has been shown to impact regeneration of the intestinal epithelium after injury, and succinate, which has been shown to increase susceptibility to Clostridium difficile infection (44, 45). Metabolic control by EIIA^Glc^ also contributes to the intestinal environment. EIIA^Glc^ modulates levels of cecal fluid polyamines such as *N*-acetyl-spermine and *N*-acetyl-putrescine, which are known to play a role in cellular proliferation and differentiation, and *S*-adenosylmethionine, a key intermediate in the methionine cycle. This cycle participates in methylation reactions essential for many biosynthetic reactions; RNA, DNA, and protein methylation; cell proliferation; and epigenetic regulation. While the infant rabbit model as currently implemented does not lend itself to studies of the long-term metabolic effects of cholera on the host, we propose that our findings justify the development of such models.

The most common sequelae of diarrheal disease are malnutrition, developmental delay, and stunting (46–48). This argues for an expanded view of virulence which includes pathogen metabolism within the intestine. By understanding pathogen metabolic pathways that are active in the host intestine at the molecular level, it may be possible to design dietary interventions that promote host metabolic health and normal host development in the aftermath of diarrheal infection.

## MATERIALS AND METHODS

### Strains, plasmids, and media.

Strains and plasmids used in these experiments are listed in Table S1 in the supplemental material. Vibrio cholerae was cultured at 27°C in Luria-Bertani (LB) broth or a previously described minimal medium (MM) supplemented with the indicated carbon source (Sigma) at a 0.5% (wt/vol) concentration (15). Where noted, the medium was also supplemented with streptomycin (100 µg/ml), ampicillin (100 µg/ml), or l-arabinose (0.04%, wt/vol).

10.1128/mBio.00858-18.9 TABLE S1  Strains and plasmids. Download TABLE S1, PDF file, 0.1 MB. Copyright © 2018 Vijayakumar et al. 2018 Vijayakumar et al. This content is distributed under the terms of the Creative Commons Attribution 4.0 International license.

### Cloning and Gibson cloning assembly.

Chromosomal modifications of the gene encoding EIIA^Glc^ were carried out as follows. The gene encoding the entire EIIA^Glc^ or an N-terminal truncation was amplified using PCR and ligated into pBAD-TOPO (Life Technologies). The nucleotide sequence was subsequently confirmed by sequencing. These sequences were then amplified from pBAD-TOPO using primers that included the V5 and 6×His affinity tags encoded in the pBAD vector. Additional fragments corresponding to the chromosomal regions 500 bp upstream and downstream of EIIA^Glc^ were also amplified using primers that included a 25-bp overlap with the EIIA^Glc^ or Δ16 EIIA^Glc^ nucleotide sequence to permit Gibson assembly. These three fragments were ligated with the pWM91 suicide vector by Gibson assembly per the manufacturer’s instructions (New England Biolabs). These constructs were then incorporated into the chromosome by homologous recombination as previously described (49). For construction of 1X-MreB, 2X-MreB, and MinD fusions to EIIA^Glc^, a similar procedure was used except that the nucleotide sequences of these AHs were codon optimized for V. cholerae and then incorporated into the primers used for amplification of Δ16 EIIA^Glc^. For strains expressing neon green, the amino acid sequence of neon green was codon optimized for V. cholerae, synthesized as a gene block (Integrated DNA Technologies [IDT]) with or without the N-terminal EIIA^Glc^ AH, and cloned into the pBAD-TOPO expression vector (Life Technologies).

### Cellular fractionation.

To separate membrane and cytoplasmic fractions of cells, V. cholerae was grown in LB broth and harvested by centrifugation in early stationary phase at an optical density at 600 nm (OD_600_) of 0.8. The resulting pellet was resuspended in 10 ml of phosphate-buffered saline (PBS) containing a protease inhibitor cocktail (Sigma) and 5 mM EDTA. Lysozyme was added to a final concentration of 1 mg/ml, and cells were incubated on ice for 30 min. DNase was then added to the lysate to reach a final concentration of 100 µg/ml, and the cells were incubated on ice for an additional 30 min. The lysate was sonicated, and cells remaining intact after this treatment were removed by passing the mixture through an 0.2-µm syringe filter. The filtrate was ultracentrifuged at 30,000 × *g* for 1 h to pellet the membrane fraction, leaving the cytoplasmic contents in the supernatant. The pellet was washed with 10 ml of PBS and ultracentrifuged again to remove residual cytoplasmic proteins.

### Coimmunoprecipitation.

For the planktonic fraction, V. cholerae was cultured with shaking at 30°C in 10 ml of LB broth until early stationary phase (OD_600_ of 0.8). For the biofilm fraction, 10 ml of LB broth was inoculated with V. cholerae in a 100-mm petri dish and incubated overnight at 30°C. Planktonic cells were removed by aspiration, and biofilm cells were resuspended in PBS. MshH and AC with C-terminal tandem affinity purification (TAP) tags were detected using anti-calmodulin binding protein antibodies (Life Technologies) (5). Cells were harvested by centrifugation, resuspended in 1 ml of buffer 1 (20 mM K-HEPES, pH 7.9, 50 mM KCl, 0.5 mM dithiothreitol [DTT], 10% glycerol, and protease inhibitor cocktail [Sigma]), and lysed by sonication. Intact bacteria were removed by centrifugation. The lysate was resuspended in an equal volume of equilibration buffer provided with the HisPur cobalt resin (Thermo) and incubated with the cobalt resin for 30 min at 4°C. The resin was then washed, and His-tagged proteins were eluted per the manufacturer’s instructions. The wash and eluent were treated as the unbound and bound fractions, respectively. Thirty microliters of wash or eluent was combined with an equal volume of Laemmli buffer (Bio-Rad) containing β-mercaptoethanol and boiled. Ten microliters of this mixture was loaded and separated on a 4 to 20% SDS gel and then transferred to a polyvinylidene difluoride membrane for Western analysis.

### Separation of phosphorylated EIIA^Glc^ by gel electrophoresis.

The protocol implemented here to preserve phosphorylated proteins in cell lysates was adapted from the work of Park et al. (50). Briefly, 2 ml of culture grown in LB supplemented with specified sugar was pelleted and resuspended in 0.2 ml PBS. To this, 20 µl of 10 M NaOH was added and vortexed for 10 s. Then, 180 µl of 3 M sodium acetate (pH 5.5) and 1 ml of ethanol were added. Samples were chilled at −80°C for 1 h, thawed, and centrifuged to pellet precipitated proteins. The pellet was washed in 70% ethanol, resuspended in 30 µl SDS loading buffer, and separated on a Phos-tag acrylamide gel prepared according to the manufacturer’s instructions (NARD Institute). Phos-tag acrylamide retards migration of phosphorylated proteins. Both phosphorylated and nonphosphorylated EIIA^Glc^ alleles were detected by Western blotting using an anti-V5 affinity tag antibody.

### Sugar transport assay.

MM supplemented with the indicated carbon source (0.5%, wt/vol) as well as a mixture of the pH indicators thymol blue (0.06 g/liter; Sigma) and bromothymol blue (0.06 g/liter; Sigma) was dispensed into a 384-well plate (30 µl) and inoculated with the indicated strain (10 µl). The OD_615_ was measured every 30 min for 18 h to monitor the rate of sugar transport as previously described (11). In parallel, growth in MM without pH indicators was monitored by measuring the OD_655_.

### Biofilm quantification.

Biofilm measurements were performed as previously described (2). Briefly, 300 µl of MM supplemented with the indicated carbon source was inoculated with V. cholerae strains at an initial OD_600_ of 0.05 in borosilicate tubes. Cells were incubated overnight at 27°C. The next day, 100 µl of planktonic cells was collected by piercing the biofilm at the air-liquid interface. The remaining planktonic cells were discarded. To measure surface-attached cells, 300 µl of PBS and a small number of glass beads were added to the tube and incubated for 30 min to 1 h. The biofilm was then disrupted by vortexing vigorously for 30 s, and 100 µl of this suspension was collected for measurement. The readings were taken using a 96-well microplate reader at 655 nm. Four biological replicates were performed for each condition.

### *csrB* degradation assays.

The indicated strains were grown to early log phase (OD_600_ of <0.4). Rifampin was added to a final concentration of 100 µg/ml, and 2 ml of cell culture was removed at 15-min intervals for 1 h. Total RNA was extracted from each sample using an RNeasy minikit (Qiagen) and quantified using a NanoDrop instrument (Thermo Scientific). One microgram of RNA was then separated on a glyoxal gel, transferred to a nylon membrane (Roche) by capillary transfer, and hybridized overnight with a *csrB*-specific oligonucleotide probe generated using the digoxigenin (DIG) labeling kit (Roche). Membranes were processed using the DIG Wash and Block buffer set (Roche) per the manufacturer’s instructions and developed using the anti-DIG antibody and CDP-Star chemiluminescent substrate (Roche).

### Infant rabbit infection and pathological analysis.

Briefly, V. cholerae was cultured overnight at 30°C and resuspended in sodium bicarbonate buffer (2.5 g in 100 ml, pH 9) to yield 10^10^ organisms in a 200-µl volume. One- to 2-day-old New Zealand White rabbit kits (Covance) were fed 50 mg/kg of weight of cimetidine 3 h prior to infection. Rabbits were inoculated using a 3.5 French red rubber catheter. Rabbits were monitored for 18 h postinfection for onset of cholera-like symptoms of diarrhea and dehydration. Once the kits started showing symptoms, they were sacrificed by administration of ketamine and xylazine via the intraperitoneal route. After confirming death, the terminal ileum, cecum, and cecal fluid were collected. A portion of the tissue was homogenized in PBS and spread on agar plates containing streptomycin to assess V. cholerae colonization. Another portion of the tissue was fixed in 10% formalin, embedded in paraffin, and stained to assess histopathology. A third portion of tissue was placed in RNAlater for reverse transcription-quantitative PCR (qRT-PCR) analysis. Colonization was unsuccessful in 2/9 rabbits gavaged with wild-type V. cholerae, 2/8 gavaged with a Δ16 EIIA^Glc^ mutant, and 1/9 rabbits gavaged with a ΔEIIA^Glc^ mutant.

### Ethics statement.

Animal experiments were performed in accordance with standards outlined in the National Research Council’s *Guide for the Care and Use of Laboratory Animals* (51) and Boston Children’s Hospital’s public health service assurance. The protocol was approved by the Boston Children’s Hospital Institutional Animal Care and Use Committee (IACUC) appointed to review proposals for research involving vertebrate animals (protocol number 14-06-2706). All efforts were made to minimize distress, pain, and suffering.

### Metabolomics.

Three hundred microliters of rabbit cecal fluid was used for metabolomics analysis with the exception of fluid from one rabbit infected with a Δ16 EIIA mutant because only 185 µl was available. Cecal fluid was centrifuged twice for 10 min at 5,000 × *g* to remove particulates, cells, and bacteria. Methanol was added to yield an 80% methanol solution, which was incubated for 6 h at −80°C and then centrifuged for 10 min at 14,000 × *g*. For LB metabolomics, 1 ml of an overnight culture of V. cholerae was centrifuged and then passed through an 0.2-µm syringe filter to remove whole cells and particulates. Methanol was added to the resulting supernatant to yield an 80% methanol solution. These solutions were desiccated at ambient temperature in a SpeedVac concentrator (Savant). The resulting pellet was stored at −80°C while awaiting analysis.

For LC-MS/MS, metabolite pellets were resuspended in 20 µl LC-MS-grade water, and 5 µl was injected over a 15-min gradient using a 5500 QTrap triple quadrupole mass spectrometer (AB Sciex) coupled to a Prominence ultrafast LC (UFLC) high-performance liquid chromatography (HPLC) system (Shimadzu) via SRM (selected reaction monitoring) of a total of 287 SRM transitions using positive- and negative-polarity switching corresponding to 258 unique endogenous water-soluble metabolites. The dwell time was 3 ms per SRM, resulting in ~10 to 14 data points acquired per detected metabolite. Samples were separated using an Amide XBridge HPLC hydrophilic interaction liquid chromatographic (HILIC) column (3.5 µm; 4.6-mm inner diameter [i.d.] by 100-mm length; Waters) at 300 µl/min. Gradients were run starting from 85% buffer B (HPLC-grade acetonitrile) to 40% B from 0 to 5 min and 40% B to 0% B from 5 to 16 min, 0% B was held from 16 to 24 min, the gradient was run from 0% B to 85% B from 24 to 25 min, and 85% B was held for 7 min to reequilibrate the column. Buffer A was comprised of 20 mM ammonium hydroxide-20 mM ammonium acetate (pH 9.0) in 95:5 water-acetonitrile. Peak areas from the total ion current for each metabolite SRM transition were integrated using MultiQuant version 2.1 software (AB Sciex) via the MQ4 peak integration algorithm using a minimum of 8 data points with a 20-s retention time window.

### qRT-PCR.

For RNA isolation from rabbit ileum, approximately 0.5 cm of ileum was dissected and incubated with RNAlater solution (Thermo Fisher) overnight at 4°C. The next day, excess RNAlater was removed, and tissues were stored at −80°C. For RNA isolation, tissues were thawed and resuspended in buffer RLT (Qiagen RNeasy kit) and disrupted with zirconia beads in a mini-bead beater (BioSpec Products). The homogenized lysate was used for RNA isolation using RNeasy minicolumns. RNA was subjected to on-column DNase I digestion (Qiagen) and Turbo DNase digestion (Life Technologies) to ensure removal of genomic DNA. RNA was quantified in a NanoDrop, and 500 ng of total RNA was used for reverse transcription using the SuperScript III first-strand synthesis set (Life Technologies). cDNA was prepared and used for qRT-PCR in a Step One Plus thermocycler (Applied Biosystems) using a Sybr green probe (Bio-Rad). Fold induction was calculated using the threshold cycle (ΔΔ*C*_*T*_) method using *clpX* as a housekeeping gene, and values were normalized using wild-type V. cholerae cultured in LB broth.

### Data analysis.

HeliQuest was used to identify amphipathic helices at the N termini of EIIA^Glc^ protein homologs (9). Clustal Omega was used for sequence alignments (52).

For Western blot analysis, band intensities were quantified by densitometry using ImageJ (53). For membrane fractionation experiments, bands associated with EIIA^Glc^ and RNA polymerase in the membrane and cytoplasmic fractions were quantified. The ratio of membrane (M) and cytoplasmic (C) band intensities normalized to RNA polymerase was taken. A relative membrane association index (MAI) was calculated for each fractionation representing (M/C)^test^/(M/C)^standard^. In Fig. 1 and 4, the ratio taken is indicated for each quantification. For partner interaction analysis, bands associated with partners in the lysate (L) and immunoprecipitate (B) were quantified using ImageJ, and a partner association index (PAI) was calculated representing the ratio of B/L for Δ16 EIIA^Glc^ divided by that for FL.

For metabolomics, hierarchical clustering of peak intensity values for each metabolite was performed using a Euclidean distance algorithm within Morpheus software from the Broad Institute (54). Intensities were mapped to colors using the minimum and maximum of each row independently with red representing values higher than the mean and blue representing values lower than the mean.

GraphPad Prism software (version 7) was used for graphing and statistical analysis. For biofilm assays, bars represent the mean and error bars represent the standard deviation from four independent biological replicates. Three independent biological replicates were performed for all transport assays and metabolomic analyses. Tests used to determine statistical significance are described in the figure legends. A *P* value <0.05 was considered statistically significant.
