# Supplementary material for: Removal of a Membrane Anchor Reveals the Opposing Regulatory Functions of Vibrio cholerae Glucose-Specific Enzyme IIA in Biofilms and the Mammalian Intestine
Source: mBio. 2018 Sep 4;9(5):e00858-18. doi: 10.1128/mBio.00858-18 (PMC6123446; doi:10.1128/mBio.00858-18)
Supplement: FIG S1 [file mbo004184039sf1.pdf]

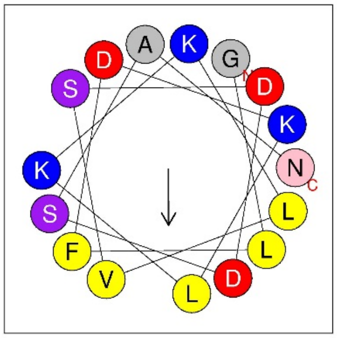

EIIA^{Glc}
µH 0.442

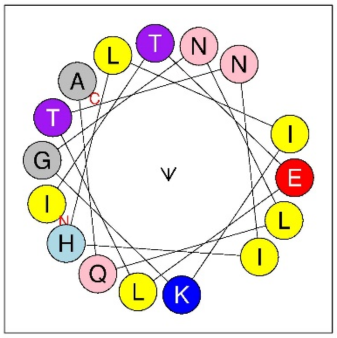

VC1820 (A)
µH 0.089

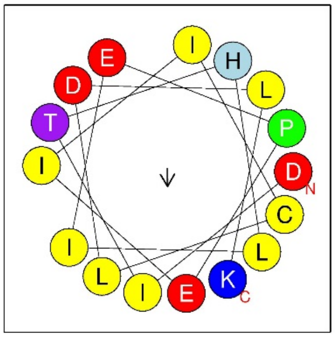

VC1822 (ABC)
µH 0.142

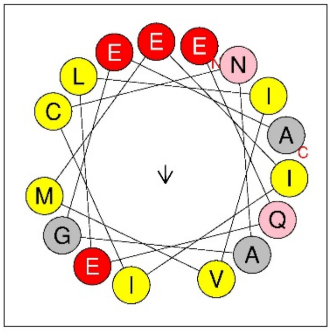

VC1283 (A)
µH 0.143

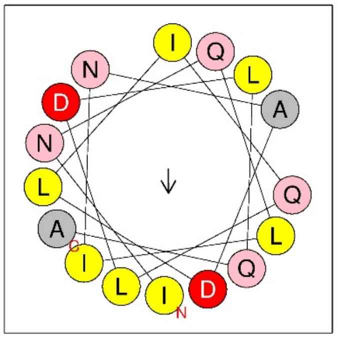

VC1826 (ABC)
µH 0.190

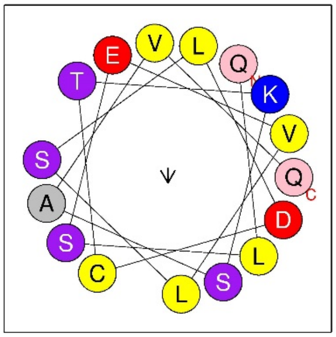

VC2531 (A)
µH 0.113

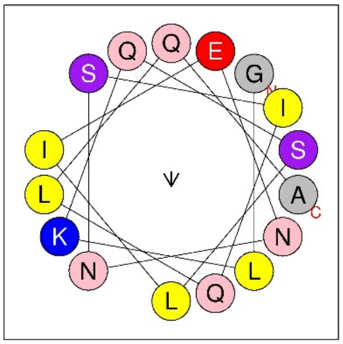

VCA0245 (A)
µH 0.109

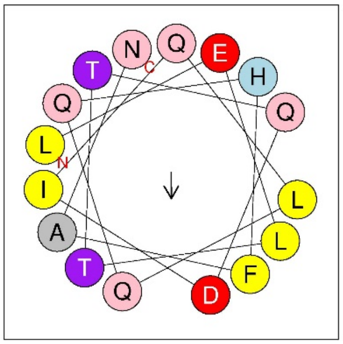

VCA0518 (A FPr)
µH 0.204

Fig S1: The hydrophobic moment of the N-terminus of EIIA^{Glc} is larger than that of other solo EIIA or EIIABC homologs of *V. cholerae*. Helical wheel projections of the first 16 amino acids of all *V. cholerae* EIIA homologs that are either alone or found at the N-terminus of a protein. Hydrophobic residues are shown in yellow. The central arrows indicate the magnitude and direction of the hydrophobic moment. The PTS Enzyme II domains found in each protein are indicated in parentheses, and the hydrophobic moment (µH) is shown below. Projections were generated with the HeliQuest software.
