# Supplementary material for: Removal of a Membrane Anchor Reveals the Opposing Regulatory Functions of Vibrio cholerae Glucose-Specific Enzyme IIA in Biofilms and the Mammalian Intestine
Source: mBio. 2018 Sep 4;9(5):e00858-18. doi: 10.1128/mBio.00858-18 (PMC6123446; doi:10.1128/mBio.00858-18)
Supplement: FIG S2 [file mbo004184039sf2.pdf]

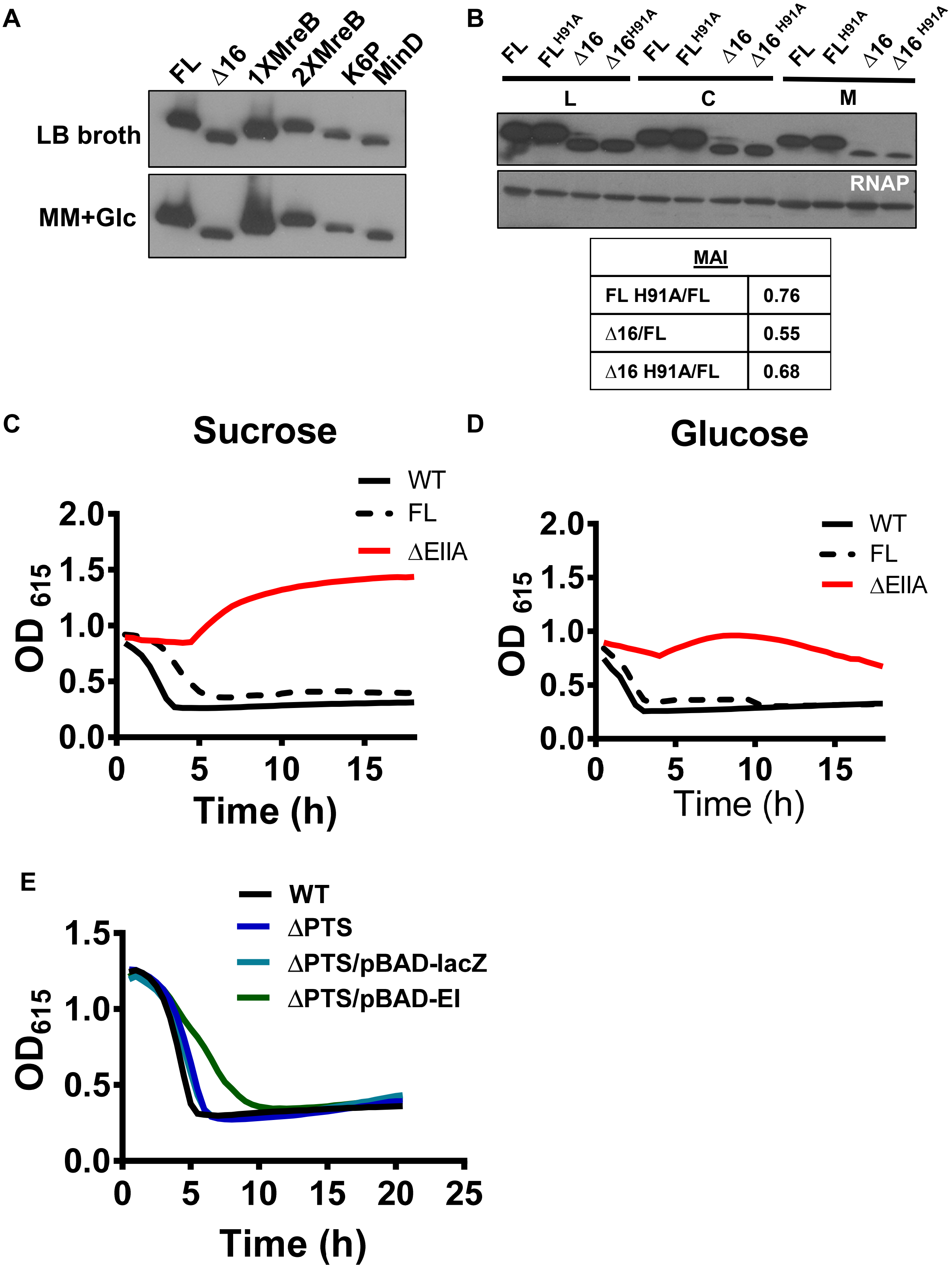
A
FL
Δ16
1XMreB
2XMreB
K6P
MinD
LB broth
MM+Glc
B
FL
FL H91A
Δ16
Δ16 H91A
L
C
M
RNAP
MAI
FL H91A/FL 0.76
Δ16/FL 0.55
Δ16 H91A/FL 0.68
C
Sucrose
WT
FL
ΔEIIA
OD 615
Time (h)
D
Glucose
WT
FL
ΔEIIA
OD 615
Time (h)
E
WT
ΔPTS
ΔPTS/pBAD-lacZ
ΔPTS/pBAD-EI
OD615
Time (h)

Fig S2: Expression, membrane association, and function of mutant EIIA^{Glc} alleles. **(A)** Western blot analysis of affinity tagged full-length EIIA^{Glc} (FL) and mutants with the first 16 amino acids removed (Δ16), one (1X MreB) or two MreB (2X MreB) amphipathic helices at the N-terminus, a K6P point mutant, and one MinD amphipathic helix at the C-terminus (MinD). **(B)** Immunoblots of the lysate (L), cytoplasmic (C), and membrane (M) fractions of *V. cholerae* strains carrying the indicated EIIA^{Glc} alleles. The membrane association index is given below. Transport of **(C)** sucrose and **(D)** glucose by *V. cholerae* with a wild-type (WT), C-terminally tagged (FL), or deleted (ΔEIIA) EIIA^{Glc} allele. **(E)** Transport of glucose by wild-type *V. cholerae* (WT) and a mutant with deletions of *EI*, *HPr*, and *EIIA^{Glc}* (ΔPTS) alone, carrying a control vector with inducible expression of β-galactosidase (pBAD-lacZ), or carrying a vector with inducible expression of *EI* (pBAD-EI).
