# Supplementary material for: Removal of a Membrane Anchor Reveals the Opposing Regulatory Functions of Vibrio cholerae Glucose-Specific Enzyme IIA in Biofilms and the Mammalian Intestine
Source: mBio. 2018 Sep 4;9(5):e00858-18. doi: 10.1128/mBio.00858-18 (PMC6123446; doi:10.1128/mBio.00858-18)
Supplement: FIG S3 [file mbo004184039sf3.pdf]

A

FL: MGLFDKLKKLVSD-EIIA^{Glc}

K6P: MGLFDPLKKLVSD-EIIA^{Glc}

1XMreB: MLKKFRGM-EIIA^{Glc}

2XMreB:MLKKFRGMFGGSEQQLKKFRGM-EIIA^{Glc}

MinD:EIIAGlc-KGFLKRLFGG

B

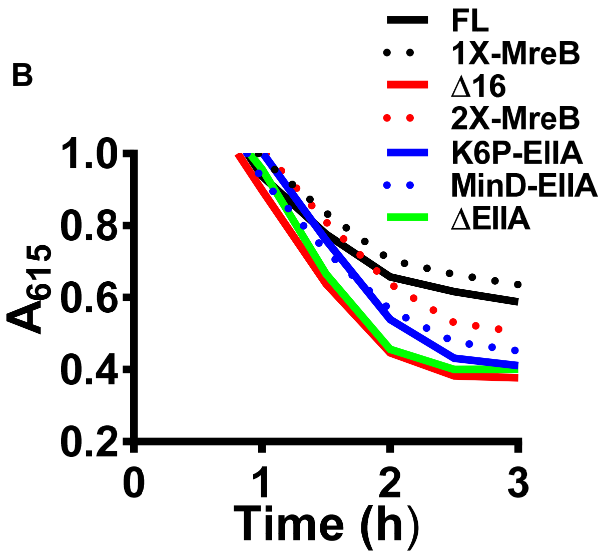

Fig S3: Maltose exclusion does not depend on AH sequence. (A) Amino acid sequences of wild-type and alternative amphipathic helices combined with Δ16-EIIA^{Glc}. **(B)** Absorbance at 615nm over time in minimal medium supplemented with maltose and α-methylglucoside is shown for strains encoding affinity tagged full-length EIIA^{Glc} (FL) and Δ16 EIIA^{Glc} (Δ16), 1X MreB-EIIA^{Glc} (1X MreB) or 2X MreB- EIIA^{Glc} (2X MreB), K6P-EIIA^{Glc} (K6P), EIIA^{Glc}-MinD (MinD), and ΔEIIA^{Glc} mutants.
