# Supplementary material for: Removal of a Membrane Anchor Reveals the Opposing Regulatory Functions of Vibrio cholerae Glucose-Specific Enzyme IIA in Biofilms and the Mammalian Intestine
Source: mBio. 2018 Sep 4;9(5):e00858-18. doi: 10.1128/mBio.00858-18 (PMC6123446; doi:10.1128/mBio.00858-18)
Supplement: FIG S4 [file mbo004184039sf4.pdf]

```
B. burgdorferi       -------MGF-------------LDFFKKTATLDLIAPISGKVMSIDKVPDEAFAEKIVG
Streptomyces         MITGAAAAGLPHAPALASDRRAAAGRNGAAHTVDIYAPLSGEIVSIEDVPDVVFAEKIVG
S. pneumoniae        -------MGL-----FDKLFGS---KENKSVEVEIYAPISGEIVNIEDVPDVVFSEKIVG
V. cholerae          -------MGL-----FDKLKKLVSDDSANAGAIDIIAPLSGEIVNIEDVPDVVFAEKIVG
E. coli              -------MGL-----FDKLKSLVSDDKKDTGTIEIIAPLSGEIVNIEDVPDVVFAEKIVG
B. pseudomallei      -----------MRRAE----ESQLKQQASHDQIVLVAPLTGPVVPLADVPDPVFSGGMFG
S. aureus            -----------MESMFK---KLFSKGKEVNKDIAIYAPLTGEYVKIEDIPDPVFAQKMMG
L. monocytogenes     --------------MFK---KFLKK----SKQEVLFAHVTGQVIALEDVPDPVFNQKMMG
                                                       : * ::*  : : .:** .*   :.*

B. burgdorferi       DGIAILPTSNELLAPCDGKIGKIFKTNHAFSLETKEGVEIFVHFGINTLNLNGKGFTRVA
Streptomyces         DGIAIAPSGSAIVAPADGTIGKIFDTDHAFSIETDSGLELFVHFGIDTVELKGQGFRRVA
S. pneumoniae        DGVAVRPIGNKIVAPVDGVIGKIFETNHAFSMESKEGVELFVHFGIDTVELKGEGFTRIA
V. cholerae          DGIAIKPAGNKMVAPVNGTIGKIFETNHAFSIESDDGVELFVHFGIDTVELKGEGFKRIA
E. coli              DGIAIKPTGNKMVAPVDGTIGKIFETNHAFSIESDSGVELFVHFGIDTVELKGEGFKRIA
B. pseudomallei      DGIGIDPLEGRLLAPCAGVVSHVARTGHAVTIAADGGAEILLHIGIDTVELNGLGFTAKI
S. aureus            EGFGINPTEGEVVSPISGRVDNVFPTKHAIGLKADNGLELLVHIGLDTVQLDGEGFEVLV
L. monocytogenes     EGIAIKPETGTIVAPIDGKIIQLAETKHAFGIRTDMGQEILVHIGLETVSLNGEGFNVLV
                     :*..: *  . :::*  * : ::  * **. : :. * *:::*:*::*:.*.* **

B. burgdorferi       EEGINVKQGEVIIRLDLEYLKEHSESVITPVVIANSDEVSSIEYSFGRLENDSEYILSSS
Streptomyces         EEGQRVTRGDTIIEVDLALLEEKAKSTLTPVVISNMDALTGLTKLSGPVT----------
S. pneumoniae        QEGQSVKRGDTVIEFDLALLESKAKSVLTPIVISNMDEISCIVKKSGEVV----------
V. cholerae          EEGQSVKIGDTIIEFDLALLEEKAKSTLTPVVISNMDEIKELNKLSGSVT----------
E. coli              EEGQCVKVGDTVIEFDLPLLEEKAKSTLTPVVISNMDEIKELIKLSGSVT----------
B. pseudomallei      AEGARVAAGDLLIEFDQDAIARAAHSLVSVIAIANSDAFEVV------------------
S. aureus            SSGDEVNVGDPLVRFNLEFINKNAKSVISPIIITNSDQAA-SINIHD-------------
L. monocytogenes     SVGDKVKVGDSIVEADFDFIEKNAASTVVPMVITNSSEGKYDFDFHA-------------
                       *  *  *: ::. :   :   : * :  : *:* .

B. burgdorferi       TVLTEEIRHKISQTKPVIAGKDLVLRVKK------------------------
Streptomyces         ------------------AGRSVILRATKE-----------------------
S. pneumoniae        ------------------AGESVVLALKK------------------------
V. cholerae          ------------------VGETPILRVTK------------------------
E. coli              ------------------VGETPVIRIKK------------------------
B. pseudomallei      --------------ERAGAGRRESGRDAAARAARARRGCKCGYKCECECGRGC
S. aureus            -------------EKAVIKGETKVIDVTMN-----------------------
L. monocytogenes     -------------VTKAEAGKTEVITTNLK-----------------------
                                        *.
```

Fig S4: The sequence of the EIIA^{Glc} AH is not conserved. Clustal Omega was used to align the sequences of the EIIA^{Glc} homologs of *Borrelia burgdorferi* (B. burgdorferi), *Streptomyces* ScaeMP-e83*, Streptococcus pneumoniae* (S. pneumoniae), *Vibrio cholerae* (V. cholerae), *Escherichia coli* (E. coli), *Burkholderia pseudomallei* (B. pseudomallei), *Staphylococcus aureus* (S. aureus), and *Listeria monocytogenes* (L. monocytogenes). Red font is used to highlight the amino acid separating the poorly conserved N-terminus of EIIA^{Glc} from the protein core. While the sequence of the body of EIIA^{Glc} is conserved, the N terminus varies.
