# Supplementary material for: Removal of a Membrane Anchor Reveals the Opposing Regulatory Functions of Vibrio cholerae Glucose-Specific Enzyme IIA in Biofilms and the Mammalian Intestine
Source: mBio. 2018 Sep 4;9(5):e00858-18. doi: 10.1128/mBio.00858-18 (PMC6123446; doi:10.1128/mBio.00858-18)
Supplement: FIG S5 [file mbo004184039sf5.pdf]

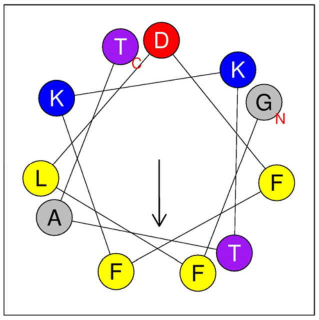

Borrelia burgdorferi
$_{1}$GFLDFFKKTAT$_{11}$
μH 0.468

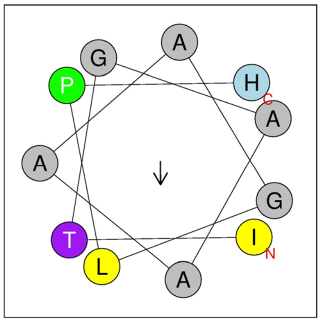

***Streptomyces* ScaeMP-e83**
$_{2}$ITGAAAAGLPH$_{12}$
μH 0.195

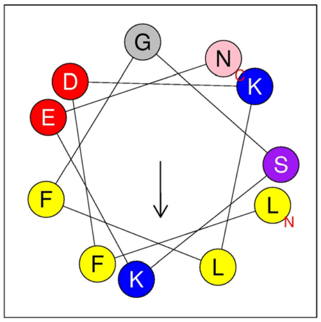

Streptococcus pneumoniae
$_{3}$LFDKLFGSKEN$_{13}$
μH 0.465

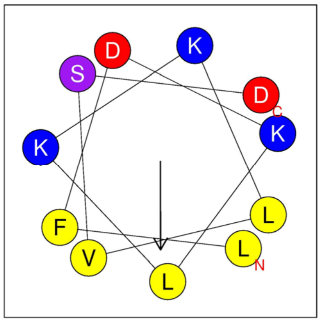

Vibrio cholerae
$_{3}$LFDKLKKLVSD$_{13}$
μH 0.737

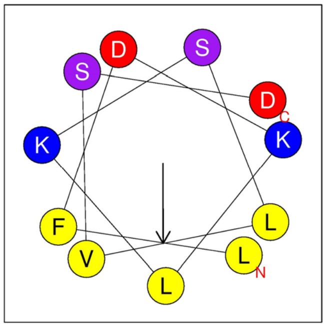

Escherichia coli
$_{3}$LFDKLKSLVSD$_{13}$
μH 0.655

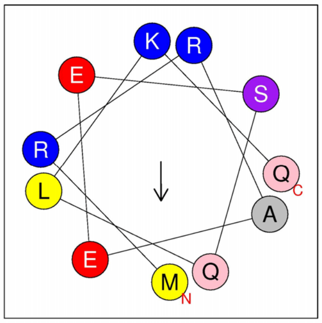

Burkholderia pseudomallei
$_{1}$RRAEESQLKQQ$_{11}$
μH 0.195

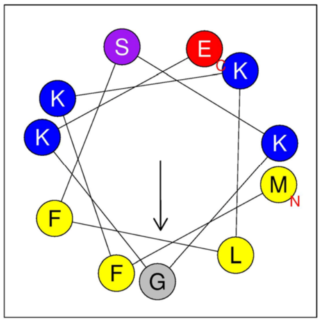

Staphylococcus aureus
$_{3}$MFKKLFSKGKE$_{13}$
μH 0.576

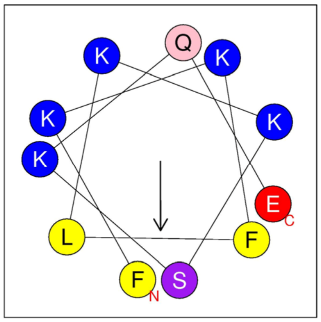

Listeria monocytogenes
$_{1}$FKKFLKKSKQE$_{11}$
μH 0.578

Fig S5: In spite of poor N-terminal amino acid sequence conservation, the EIIA^{Glc} proteins of unrelated bacteria have a predicted N-terminal amphipathic helix. Heliquest software was used to identify amphipathic helices in the poorly conserved N-termini of EIIA^{Glc} proteins from the indicated organisms. In each case except that of the distantly related *Streptomyces* and *Burkholderia* species, an 11-amino acid amphipathic helix whose sequence is listed below the bacterial name, was found at the very beginning of the protein. The hydrophobic moment μH for each helix is given below.
