# Supplementary material for: Removal of a Membrane Anchor Reveals the Opposing Regulatory Functions of Vibrio cholerae Glucose-Specific Enzyme IIA in Biofilms and the Mammalian Intestine
Source: mBio. 2018 Sep 4;9(5):e00858-18. doi: 10.1128/mBio.00858-18 (PMC6123446; doi:10.1128/mBio.00858-18)
Supplement: FIG S6 [file mbo004184039sf6.pdf]

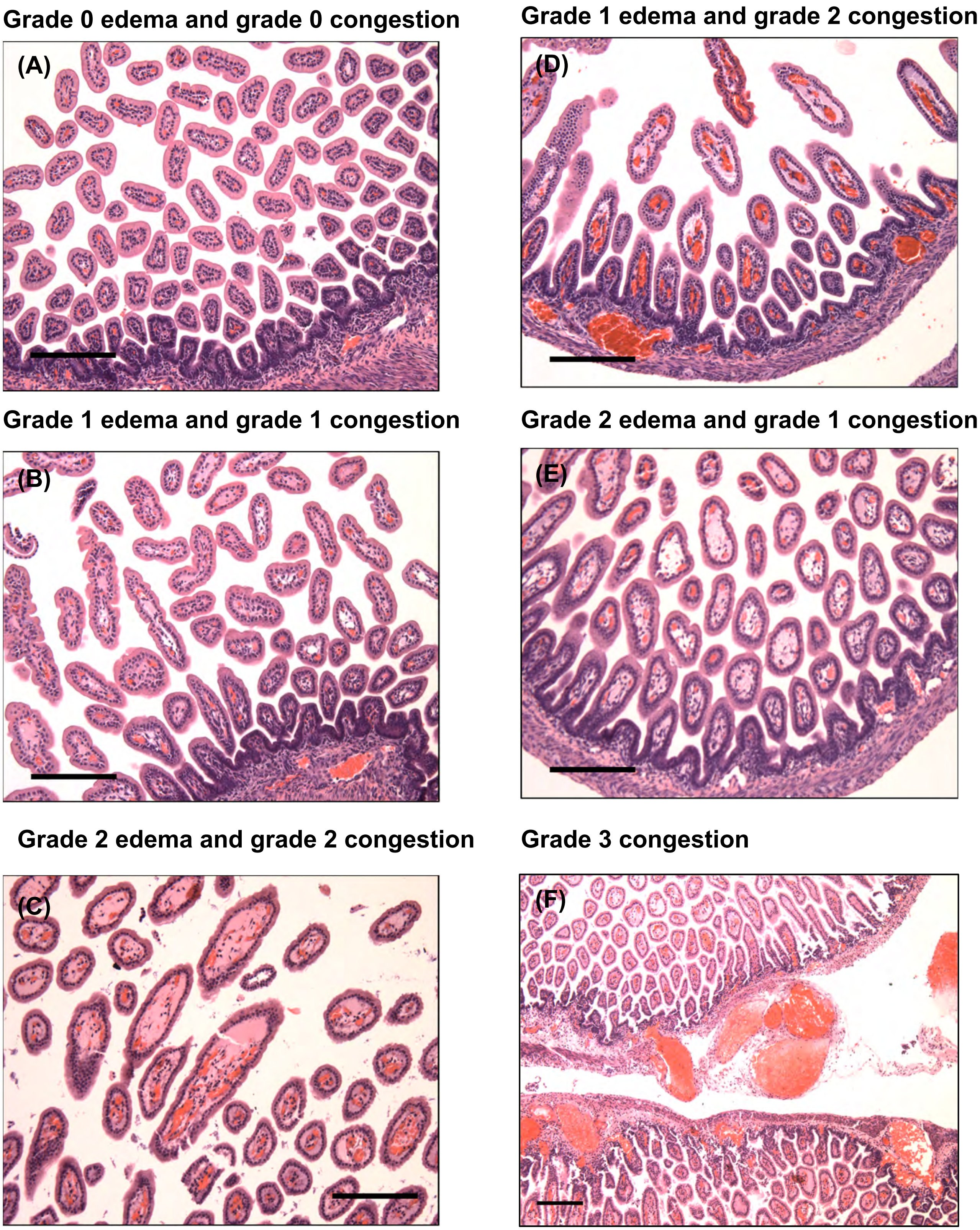

Fig S6: Scoring rubric for intestinal congestion and edema: Hematoxylin and Eosin-stained sections of the infant rabbit terminal ileum harvested at 22 h post *V. cholerae* infection showing grades 1 and 2 edema and grades 1-3 capillary congestion as noted. Pictures were taken at 20X magnification. Scale bars represent 500μm in A, B, C, D, E and 1mm in F.
