# Supplementary material for: Removal of a Membrane Anchor Reveals the Opposing Regulatory Functions of Vibrio cholerae Glucose-Specific Enzyme IIA in Biofilms and the Mammalian Intestine
Source: mBio. 2018 Sep 4;9(5):e00858-18. doi: 10.1128/mBio.00858-18 (PMC6123446; doi:10.1128/mBio.00858-18)
Supplement: FIG S7 [file mbo004184039sf7.pdf]

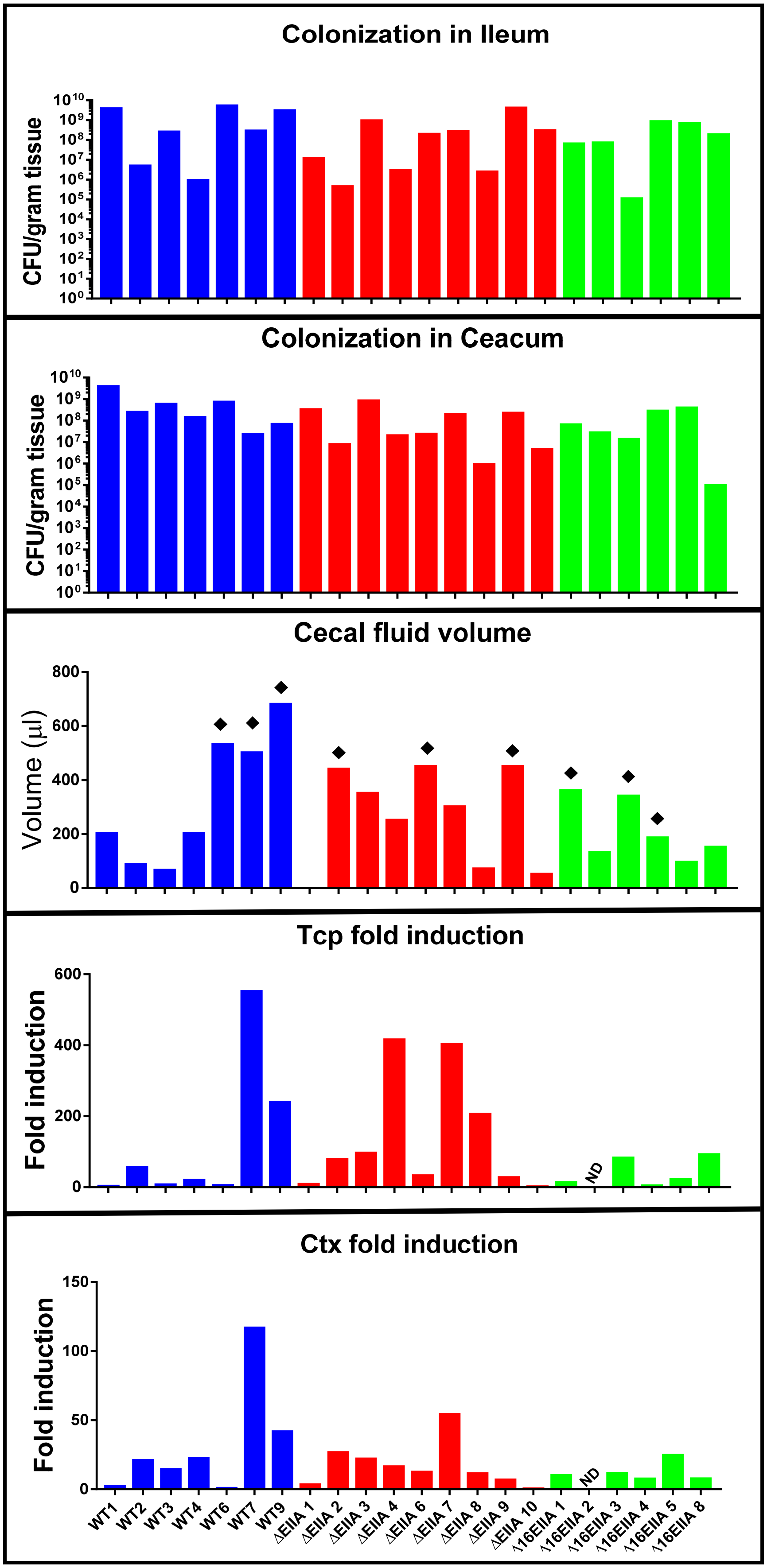

Fig S7: Characteristics of individual infant rabbits from which cecal fluid was harvested for metabolomic analysis*.* Panels show colonization, cecal fluid accumulation, *ctx* transcription, and *tcpA* transcription for individual infant rabbits. Diamonds denote infant rabbits from which cecal fluid was harvested for metabolomics studies. ND indicates not done. WT 1-5, ΔEIIA 1-2, and Δ16 1-2 came from litter 1. WT 6-9, ΔEIIA 3-10, and Δ16 3-8 came from litter 2.
