# Supplementary material for: Removal of a Membrane Anchor Reveals the Opposing Regulatory Functions of Vibrio cholerae Glucose-Specific Enzyme IIA in Biofilms and the Mammalian Intestine
Source: mBio. 2018 Sep 4;9(5):e00858-18. doi: 10.1128/mBio.00858-18 (PMC6123446; doi:10.1128/mBio.00858-18)
Supplement: FIG S8 [file mbo004184039sf8.pdf]

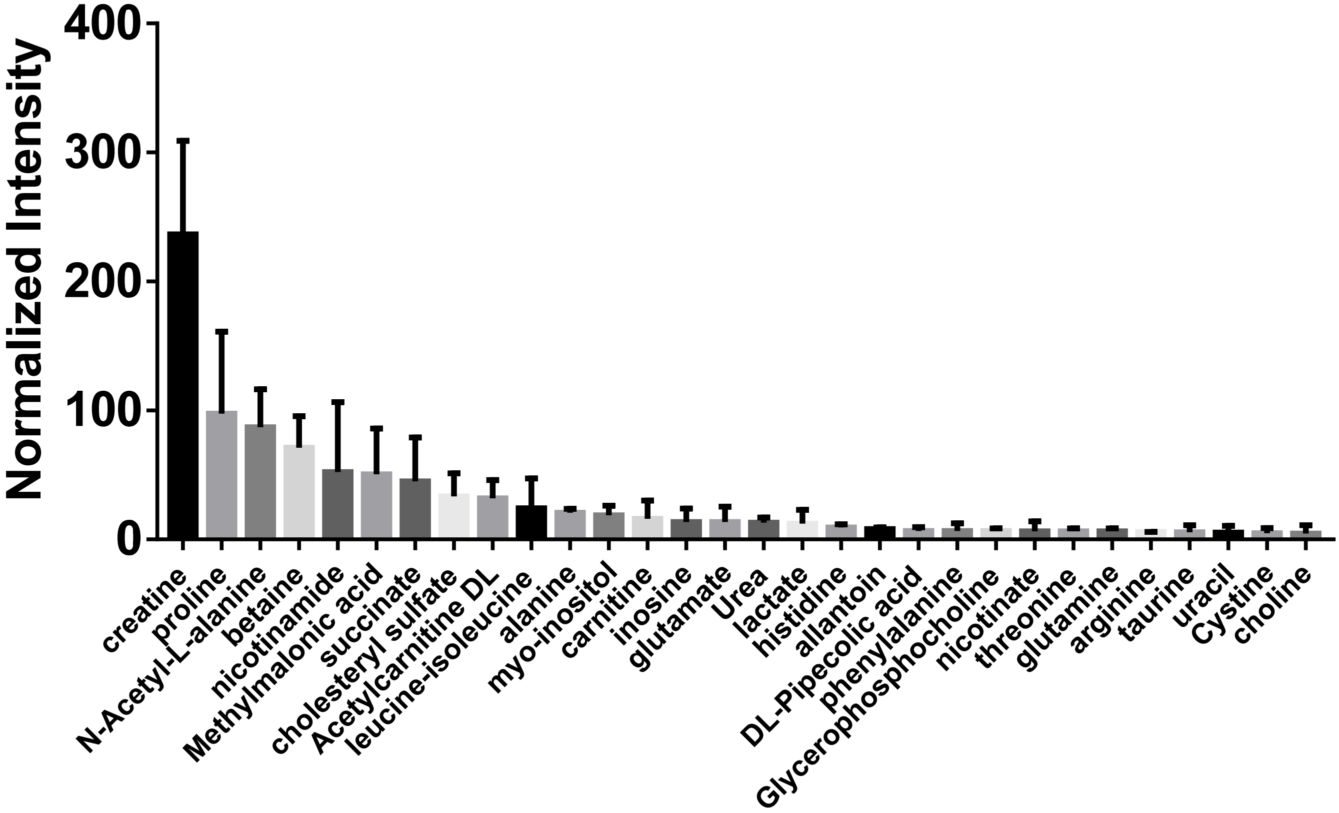

Fig S8: Thirty most abundant metabolites in cecal fluid of infant rabbits infected with wild-type V. cholerae.
