# Supplementary material for: Removal of a Membrane Anchor Reveals the Opposing Regulatory Functions of Vibrio cholerae Glucose-Specific Enzyme IIA in Biofilms and the Mammalian Intestine
Source: mBio. 2018 Sep 4;9(5):e00858-18. doi: 10.1128/mBio.00858-18 (PMC6123446; doi:10.1128/mBio.00858-18)
Supplement: TABLE S1 [file mbo004184039st1.pdf]

Table S1: Strains and plasmids

Strain	Description	Reference
E. coli SM10λpir	thi thr leu tonA lacY supE recA::RP4-2-Tc::Mu (λpirR6K) Kmr	[1]
Vibrio cholerae strains		
PW357	MO10 lacZ::vpsLp -->lacZ Sm^r	[2]
PW836	MO10 lacZ::vpsLp -->lacZ $\Delta EIIA^{Glc}$ Sm^r	[3]
PW985	MO10 lacZ::vpsLp -->lacZ ΔEI ΔHPr $\Delta EIIA^{Glc}$	[3]
PW1875	MO10 lacZ::vpsLp -->lacZ $\Delta 16EIIA^{Glc}$ Sm^r	This study
PW1876	MO10 lacZ::vpsLp -->lacZ FL-$EIIA^{Glc}$ Sm^r	This study
PW1877	MO10 lacZ::vpsLp -->lacZ K6P-$EIIA^{Glc}$ Sm^r	This study
PW1878	MO10 lacZ::vpsLp -->lacZ $EIIA^{Glc}$-MinD Sm^r	This study
PW1879	MO10 lacZ::vpsLp -->lacZ 1XMreB-$EIIA^{Glc}$ Sm^r	This study
PW1880	MO10 lacZ::vpsLp -->lacZ 2XMreB-$EIIA^{Glc}$ Sm^r	This study
PW1881	MO10 lacZ::vpsLp -->lacZ FL $EIIA^{GlcH91A}$ Sm^r	This study
PW1882	MO10 lacZ::vpsLp -->lacZ Δ16 $EIIA^{GlcH91A}$ Sm^r	This study
PW1889	MO10 lacZ::vpsLp -->lacZ FL $EIIA^{Glc}$ MshH-TAPtag::pGP704 Sm^r Ap^r	This study
PW1890	MO10 lacZ::vpsLp -->lacZ $\Delta 16EIIA^{Glc}$ MshH-TAPtag::pGP704 Sm^r Ap^r	This study
PW1891	MO10 lacZ::vpsLp -->lacZ $\Delta EIIA^{Glc}$ MshH-TAPtag::pGP704 Sm^r Ap^r	This study
PW2003	MO10 lacZ::vpsLp -->lacZ $\Delta EIIA^{Glc}$ AC-TAPtag:: pGP704 Sm^r Ap^r	This study
PW2004	MO10 lacZ::vpsLp -->lacZ $\Delta 16EIIA^{Glc}$ AC-TAPtag:: pGP704 Sm^r Ap^r	This study
PW2005	MO10 lacZ::vpsLp -->lacZ FL $EIIA^{Glc}$ AC-TAPtag:: pGP704 Sm^r Ap^r	This study
PW1974	MO10 lacZ::vpsLp -->lacZ FL $EIIA^{Glc}$-V5-His $\Delta EIIBC^{Glc}$ ΔMshH Δ*cyaA*	This study

PW1975	MO10 lacZ::vpsLp -->lacZ H Δ16-EIIA^{Glc}-V5-HisΔEIIBC^{Glc}ΔMsh Δ*cyaA*	This study
PW1898	MO10 lacZ::vpsLp -->lacZ EI^{H189A} ΔEIIA^{Glc}	This study
PW1892	MO10 lacZ::vpsLp -->lacZ EI^{H189A}	This study
PW2010	MO10 lacZ::vpsLp -->lacZ Δ16 EIIA^{Glc}-V5-6XHis ΔEIIBC^{Glc}	This study
PW2015	MO10 lacZ::vpsLp -->lacZ EI^{H189A} Δ16 EIIA^{Glc}-V5-6XHis ΔEIIBC^{Glc}	This study
PW2008	MO10 lacZ::vpsLp -->lacZ EI^{H189A} Δ16EIIA^{Glc}-V5-6XHis	This study
PW2009	MO10 lacZ::vpsLp -->lacZ EI^{H189A} FL EIIA^{Glc}-V5-6XHis	This study
PW2006	MO10 lacZ::vpsLp -->lacZ Δ16 EIIA^{Glc}-V5-His Δ*mlc*	This study
PW993	MO10 lacZ::vpsLp -->lacZ Δ*mlc*	[4]
Plasmids for complementation		
PW1464	pBAD-TOPO encoding *lacZ*	Invitrogen
PW1884	pBAD-TOPO encoding FL-EIIA^{Glc} -V5-His	This study
PW1883	pBAD-TOPO encoding Δ16EIIA^{Glc}-V5-6XHis	This study
PW1885	pBAD-TOPO encoding one MreB amphipathic helix fused to Δ16-EIIA^{Glc}-V5-6XHis	This study
PW1886	pBAD-TOPO encoding two MreB amphipathic helices (2XMreB) fused to Δ16 EIIA^{Glc}-V5-6XHis	This study
PW1887	pBAD-TOPO encoding FL EIIA^{GlcK6P}-V5-6XHis	This study
PW1888	pBAD-TOPO encoding Δ16 EIIA^{Glc} -MinD-V5-6XHis	This study
PW2000	pBAD-TOPO carrying the EIIA^{Glc} amphipathic helix fused to neon green-V5-6XHis	This study
PW1999	pBAD Neon Green-V5 -6XHis	This study
PW967	pBAD-TOPO encoding EI (Enzyme I)	[3]
Plasmids used for chromosomal recombination		

pWM91	oriR6K mobRP4 lacI pTac tnp mini-Tn10(Km) Km^r Ap^r	[5]
pWM91 FL $EIIA^{Glc}$	Plasmid used to recombine FL $EIIA^{Glc}$ onto the chromosome	This study
pWM91 FL $EIIA^{Glc}$	Plasmid used to recombine∆16 $EIIA^{Glc}$-V5-6XHis onto the chromosome	This study
pWM91 1XMreB-$EIIA^{Glc}$	Plasmid used to recombine 1XMre-$EIIA^{Glc}$-V5-6XHis onto the chromosome	This study
pWM91 2XMreB-$EIIA^{Glc}$	Plasmid used to recombine 2XMreB-$EIIA^{Glc}$-V5-6XHis onto the chromosome	This study
pWM91 K6P-$EIIA^{Glc}$	Plasmid used to recombine $EIIA^{GlcK6P}$-V5-6XHis onto the chromosome	This study
pWM91 $EIIA^{Glc}$-MinD	Plasmid used to recombine $EIIA^{Glc}$-MinD-V5-6XHis onto the chromosome	This study
pGP704::VC0398-TAP	Plasmid used to generate MshH-TAP on the chromosome	[6]
pGP704::cyaA-TAP	Plasmid used to generate AC-TAP on the chromosome	[6]
pWM91 EI H189A	Plasmid used to recombine EI^{H189A} onto the chromosome	This study

Supplementary References

1 Miller, V. L. & Mekalanos, J. J. A novel suicide vector and its use in construction of insertion mutations: osmoregulation of outer membrane proteins and virulence determinants in Vibrio cholerae requires toxR. *J Bacteriol* **170**, 2575-2583 (1988).

2 Haugo, A. J. & Watnick, P. I. *Vibrio cholerae* CytR is a repressor of biofilm development. *Mol Microbiol* **45**, 471-483, doi:3023 [pii] (2002).

3 Houot, L. & Watnick, P. I. A novel role for enzyme I of the Vibrio cholerae phosphoenolpyruvate phosphotransferase system in regulation of growth in a biofilm. *J Bacteriol* **190**, 311-320, doi:10.1128/JB.01410-07 (2008).

4 Pickering, B. S., Lopilato, J. E., Smith, D. R. & Watnick, P. I. The transcription factor Mlc promotes Vibrio cholerae biofilm formation through repression of phosphotransferase system components. *J Bacteriol* **196**, 2423-2430, doi:10.1128/JB.01639-14 (2014).

5 Metcalf, W. W. *et al.* Conditionally replicative and conjugative plasmids carrying lacZ alpha for cloning, mutagenesis, and allele replacement in bacteria. *Plasmid* **35**, 1-13, doi:10.1006/plas.1996.0001 (1996).

6 Pickering, B. S., Smith, D. R. & Watnick, P. I. Glucose-specific enzyme IIA has unique binding partners in the vibrio cholerae biofilm. *MBio* **3**, e00228-00212, doi:10.1128/mBio.00228-12 (2012).
